# Time-resolved transcriptomic analysis reveals key regulatory genes and auxin-responsive networks underlying axillary bud branching in *Hippophae rhamnoides*

**DOI:** 10.3389/fpls.2026.1746947

**Published:** 2026-03-03

**Authors:** Guozhen Yan, Qinggang Wei, Kaiwen Tan, Jin Lei, Junyang Wang, Cheng Tang, Feng Shi

**Affiliations:** 1 College of Life Science, Shihezi University, Shihezi, China; 2 College of Agriculture, Shihezi University, Shihezi, China; 3 College of Mechanical and Electrical Engineering, Shihezi University, Shihezi, China; 4 Xinjiang Silk Road Sea Buckthorn Technology Co., Ltd., Tacheng, China; 5 College of Urban and Environmental Sciences, Shihezi University, Shihezi, China

**Keywords:** auxin signaling, axillary bud development, branching regulation, hub genes, *Hippophae rhamnoides*, transcriptomics, WGCNA

## Abstract

The development of axillary buds into branches fundamentally shapes plant architecture, yet how this process is transcriptionally coordinated across developmental stages in woody perennials remains incompletely understood. Using *Hippophae rhamnoides* (sea buckthorn) as a woody perennial model, we integrated stage-resolved transcriptomic profiling across three axillary bud developmental stages with co-expression network analysis and experimental validation to characterize the regulatory landscape underlying bud activation and branch elongation. Stage-specific expression clustering revealed distinct transcriptional programs associated with developmental transitions: an early-activation gene cluster (Cluster 8, 2003 genes) was enriched in auxin signaling components and transcriptional regulators, reflecting rapid transcriptional reprogramming during bud release from dormancy. In parallel, weighted gene co-expression network analysis (WGCNA) identified key modules (e.g., blue and turquoise) containing hub genes involved in cell proliferation, metabolic adjustment, and stress-related processes, together forming a coordinated regulatory network supporting sustained bud outgrowth. Several candidate hub genes, including *ARF, IAA16*, and *SAUR36*, displayed expression patterns responsive to changes in apical auxin status, consistent with a putative “release-and-rescue” transcriptional pattern associated with axillary bud elongation. Collectively, these results support an integrative framework in which axillary bud activation in a woody perennial is regulated through temporally coordinated functional modules rather than bulk hormonal accumulation alone. This systems-level perspective provides molecular insight into shoot branching regulation and identifies candidate genes with potential utility for improving plant architecture in woody species.

## 1 Introduction

Plant branching architecture is a fundamental agronomic trait that critically determines canopy structure, light interception efficiency, and overall productivity in woody species (Yun et al., 2025; Li et al., 2017; Shao et al., 2022; Clark and Ma, 2023). In fruit trees and shrubs, optimal branching patterns enhance light distribution within the canopy, improve fruit quality, and facilitate mechanized harvesting, thereby increasing orchard management efficiency and economic yield (Zhen et al., 2025). *Hippophae rhamnoides L.* (sea buckthorn, hereafter *H. rhamnoides*), an ecologically and economically important woody shrub widely distributed across arid and semi-arid regions of Eurasia, plays key roles in windbreak and sand fixation, soil and water conservation, and ecological restoration. Its fruits are rich in vitamins, flavonoids, and polyphenols, offering substantial potential for functional foods and pharmaceuticals (Ma et al., 2023; Ciesarová et al., 2020). Nevertheless, under both natural and cultivated conditions, *H. rhamnoides* often exhibits excessive or insufficient branching, limiting nutrient allocation and fruit yield, which constrains its large-scale cultivation and industrial development.

Branching is a complex developmental process regulated by genetic, hormonal, and environmental factors. Among phytohormones, auxin, particularly indole-3-acetic acid (IAA), serves as a primary inhibitory signal, maintaining apical dominance and suppressing axillary bud outgrowth, whereas cytokinins (CKs) generally promote bud release. Strigolactones (SLs) act antagonistically to auxin, further restricting branching and contributing to a dynamic hormonal network that integrates metabolic and environmental cues (Domagalska and Leyser, 2011; Wang et al., 2018; Panda et al., 2018). In model plants, key genetic regulators—such as BRANCHED1 (*BRC1*) in Arabidopsis thaliana (Aguilar-Martínez et al., 2007; González-Grandío et al., 2013), MONOCULM1 (*MOC1*) in rice (Takai, 2024; Sun et al., 2010), and TEOSINTE BRANCHED1 (*TB1*) in maize (Dong et al., 2017, 2019)—act as central repressors of lateral bud activation. These genes represent evolutionarily conserved modules that regulate meristem fate and branch initiation across species. In addition, nutrient-sensing pathways, including trehalose-6-phosphate (*Tre6P*) and HEXOKINASE1 (*HXK1*) signaling, These nutrient-sensing pathways interact with hormonal cues to fine-tune branching decisions and contribute to the maintenance of apical dominance and suppression of axillary bud outgrowth (Ye et al., 2022; Kebrom and Doust, 2022; Beveridge et al., 2000; Foo et al., 2001).

Importantly, accumulating evidence indicates that auxin-mediated regulation of axillary bud outgrowth is not necessarily reflected by large changes in bulk IAA concentration. Instead, early bud activation and subsequent sustained growth can depend on auxin transport and canalization dynamics, spatial redistribution within tissues, and changes in cellular sensitivity and downstream transcriptional output (Vanneste et al., 2025). Experimental studies have demonstrated that initial bud outgrowth following decapitation can occur independently of immediate auxin export from the bud, whereas auxin transport becomes increasingly important for sustained growth and vascular reconnection at later stages (Chabikwa et al., 2019). This distinction is particularly relevant for transcriptomic analyses, as substantial transcriptional reprogramming may occur even when total tissue-level auxin abundance remains relatively stable.

In woody perennials, the regulation of branching is further complicated by perennial growth habits and repeated cycles of bud dormancy and reactivation. Unlike annual herbaceous plants, woody species experience seasonal growth arrest, dormancy maintenance, and subsequent bud break, processes that require coordinated regulation of carbohydrate metabolism, hormonal signaling, and cellular connectivity. Recent studies in tree systems emphasize that dormancy-associated transitions are governed by multilayer regulatory mechanisms that extend beyond classical models derived from annual species (Ding et al., 2024; Zhao et al., 2025). These features suggest that the temporal organization and integration of transcriptional programs during axillary bud activation in woody perennials may differ substantially from those described in herbaceous plants, yet remain insufficiently characterized.

Integrating stage-resolved transcriptomics with soft clustering approaches, such as Mfuzz, and WGCNA provides a powerful systems-level strategy for dissecting complex developmental processes (Sun et al., 2019; Campione et al., 2025; Li et al., 2019; Xu et al., 2023; Wu et al., 2022). Mfuzz enables the identification of genes with similar temporal expression trajectories, capturing gradual transcriptional changes across developmental stages, while WGCNA reveals co-expressed gene modules and hub regulators based on network topology. In woody species, such integrative approaches are particularly valuable, as axillary bud outgrowth emerges from the coordinated action of hormone signaling, energy metabolism, stress responses, and developmental reprogramming acting on partially overlapping timescales (Barbier et al., 2019).

However, despite these advances, how temporally coordinated transcriptional programs are organized during axillary bud activation in woody perennials remains poorly understood. Here, we employed high-throughput RNA sequencing to capture temporal expression dynamics during axillary bud development in *H. rhamnoides*, followed by Mfuzz clustering and WGCNA to construct co-expression networks and identify candidate hub genes. Key genes were further validated by Quantitative real-time PCR (qRT-PCR), and their responsiveness to hormonal cues was examined using decapitation and exogenous auxin treatments. By linking temporal expression patterns, co-expression modules, and hormone responsiveness, this study provides a systematic molecular framework for understanding axillary bud activation in a woody perennial species and offers valuable genetic resources for improving plant architecture and cultivation efficiency (Li et al., 2018; Shen et al., 2019).

## 2 Materials and methods

### 2.1 Experimental design and sample collection

**Figure 1:**
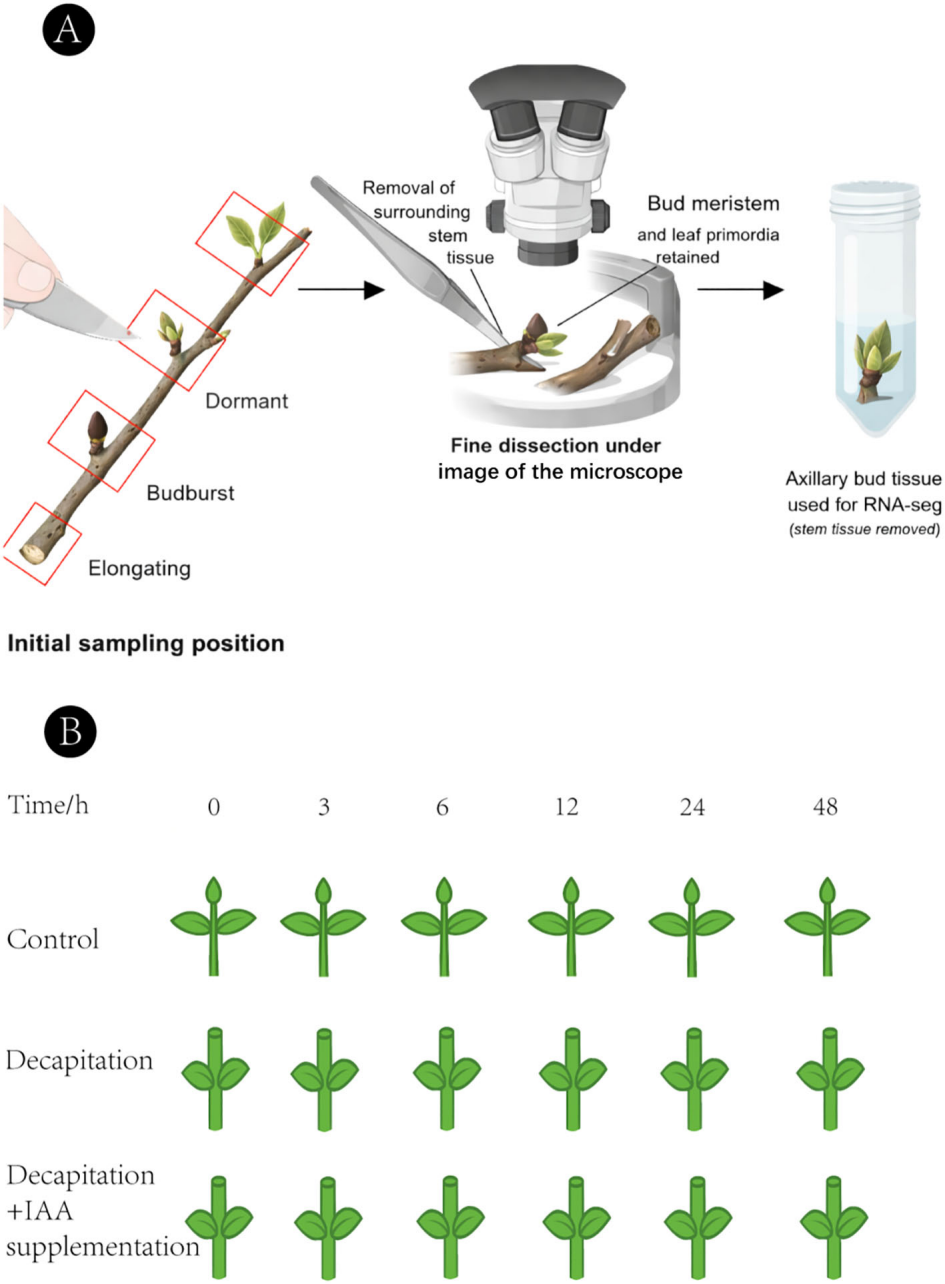
Experimental design and sampling strategy for analyzing axillary bud development in *Hippophae rhamnoides*. **(A)** The morphological phenotypes of axillary buds at three representative developmental stages sampled for transcriptome analysis: T0 (dormant bud), T1 (budburst stage, 1–2 mm), and T2 (branch elongation stage, 5–10 mm). Scale bar = 1 cm. **(B)** Schematic diagram of the decapitation and auxin treatment experiment. Apical buds of uniformly grown plants were either retained (Control), removed (Decapitation), or removed followed by immediate application of 10 μM IAA to the cut surface (Decapitation + IAA). Axillary buds were sampled at 0, 3, 6, 12, 24, and 48 hours post-treatment for temporal expression analysis.

The overall experimental design and sampling strategy are illustrated in **Figure 1**.

#### 2.1.1 Sampling of axillary bud developmental stages

Five-year-old healthy *H. rhamnoides* cv. ‘Shenqiuhong’ plants grown in Xinjiang, China, were used in this study. Five independent plants were selected as biological replicates.

Axillary buds were collected at three morphologically distinct developmental stages: T0 (dormant stage), T1 (budburst stage, 1–2 mm in length), and T2 (branch elongation stage, 5–10 mm in length), following established protocols (Ni et al., 2017; Yao et al., 2022; Tang et al., 2023). Axillary buds at different developmental stages were sampled from the same set of five plants, constituting a paired sampling design across stages.

For each plant at each developmental stage, axillary buds from multiple branches were pooled to generate one biological replicate (n = 5). Bud sampling and tissue separation were conducted based on external morphological characteristics, including bud size, shape, and visible developmental status. During dissection, only a minimal amount of immediately adjacent nodal tissue was retained when unavoidable, particularly at early developmental stages when axillary buds were small and closely associated with the stem, in order to minimize the inclusion of elongated internode or mature stem tissues.

All samples were harvested between 09:00 and 11:00 to minimize diurnal effects, immediately flash-frozen in liquid nitrogen, and stored at –80 °C until RNA extraction.

#### 2.1.2 Exogenous auxin treatment and sampling

To evaluate transcriptional responses to auxin, a decapitation and auxin replacement assay was performed. Plants were assigned to three treatments: (i) Control: apical bud retained; (ii) Decapitation: apical bud removed; (iii) Decapitation + IAA: 10 μM IAA applied immediately to the cut surface. The first pair of axillary buds below the apex, along with adjacent stem tissues, was collected at 0, 3, 6, 12, 24, and 48 hours after treatment. Three biological replicates were included for each treatment–time combination, yielding a total of 54 samples. Sample handling followed the same procedure as Section 2.1.1 (Prusinkiewicz et al., 2009; Balla et al., 2016; Beveridge et al., 2000; Shimizu-Sato et al., 2009).

### 2.2 Auxin quantification by competitive ELISA

To provide physiological context for the transcriptomic analyses and auxin-related gene expression patterns described above, endogenous indole-3-acetic acid (IAA) levels in axillary buds were quantified as described below.

Endogenous IAA levels in axillary bud tissues were quantified using a competitive enzyme-linked immunosorbent assay (ELISA) according to the manufacturer’s instructions, using a Plant Indole-3-Acetic Acid (IAA) ELISA Kit (Jiangsu Baolai Biotechnology Co., Ltd., Jiangsu, China; catalog number: A-Z0031A), with minor clarifications. Briefly, axillary bud samples collected at three developmental stages (T0, T1, and T2) were immediately frozen in liquid nitrogen and stored at −80 °C until analysis. For each biological replicate, approximately 0.1 g of frozen tissue was finely ground in liquid nitrogen and extracted according to the kit instructions. After centrifugation, the supernatant was collected for ELISA analysis.

IAA quantification was performed using a commercial competitive ELISA kit specific for plant IAA. For each sample well, 10 μL of extracted sample was mixed with 40 μL of sample diluent, resulting in a five-fold dilution (D1). To ensure that measurements fell within the linear range of the standard curve, a further dilution (D2, 1:25 final dilution) was prepared when necessary. All samples were analyzed in technical duplicates, with D1 and D2 measurements arranged symmetrically across the plate to minimize positional effects.

A standard curve was generated using six IAA standards provided with the kit, each measured in duplicate. Absorbance values were recorded at 450 nm using a microplate reader. IAA concentrations were calculated based on a logit–log regression model derived from the standard curve and were subsequently corrected for dilution factors.

Given the competitive nature of the assay, only wells exhibiting the expected inverse relationship between absorbance and IAA concentration were retained for downstream analysis. Wells showing inconsistent competitive behavior or falling outside the reliable range of the standard curve were excluded. Final IAA concentrations were expressed as nmol L^-^¹.

### 2.3 Transcriptome sequencing and data analysis

Transcriptome sequencing was performed on two sample sets: natural developmental stages (T0, T1, T2; n = 15) and IAA treatment time-course samples (n = 54).

#### 2.3.1 RNA extraction, library construction, and sequencing

Total RNA was extracted using the FastPure Plant Total RNA Isolation Kit (Vazyme, China). RNA purity and concentration were determined with a NanoDrop 2000 spectrophotometer, and integrity was assessed using an Agilent 2100 Bioanalyzer. Samples with RIN ≥ 7.0 and A260/A280 ratios of 1.8–2.2 were used for library preparation.

Strand-specific libraries were constructed from poly(A)+ mRNA enriched using oligo(dT) magnetic beads, including fragmentation, cDNA synthesis, end repair, A-tailing, adapter ligation, and PCR amplification. Library quality was verified using Qubit and qPCR. Qualified libraries were sequenced on an Illumina platform to generate 150 bp paired-end reads.

#### 2.3.2 Data quality control and read mapping

Raw reads were processed using DataFilterQC (v0.0.2; parameters: −x 5, −l 30, −n 3) to remove adapters and low-quality sequences. Clean reads were aligned to the *Hippophae rhamnoides L*. reference genome, together with the corresponding gene structure annotation files, downloaded from the China National GeneBank DataBase (CNGBdb; https://db.cngb.org/), using HISAT2 (v2.2.1; parameters: –dta-cufflinks –no-unal –un-conc-gz) (Kim et al., 2019). BAM files were sorted, merged, and indexed using SAMtools (v0.1.19) (Li et al., 2009).

#### 2.3.3 Gene expression quantification and differential expression analysis

Gene-level read counts were obtained using featureCounts (v2.0.2) (Liao et al., 2014). Differential expression analysis was performed with edgeR (v3.3.3) using TMM normalization and a negative binomial GLM (Robinson et al., 2010). Genes with |log_2_FC| ≥ 1 and FDR < 0.05 were considered differentially expressed; for comparisons with fewer than 100 differentially expressed genes (DEGs), thresholds were relaxed to |log_2_FC| ≥ 1 and p < 0.05. Transcriptome assembly and quantification were conducted using StringTie (Pertea et al., 2015) and GFF Utilities (Pertea and Pertea, 2020).

Gene models derived from the *H. rhamnoides* genome annotation were standardized and assigned unique identifiers using AGAT (Another Gff Analysis Toolkit), resulting in AGAT-based gene IDs (e.g., agat-gene-22040) used throughout this study.

#### 2.3.4 Functional annotation and enrichment analysis

Functional annotation was performed against NR, Swiss-Prot, and KEGG databases. GO and KEGG enrichment analyses were conducted using topGO (Alexa and Rahnenfuhrer, 2023) and KOBAS (Bu et al., 2021), with hypergeometric tests and Benjamini–Hochberg correction (FDR < 0.05). Transcription factors were identified using iTAK (Zheng et al., 2016), PlantTFDB, and HMMER (Potter et al., 2018).

### 2.4 Time-series clustering Analysis

Temporal gene expression dynamics were characterized using Mfuzz in R, implementing fuzzy c-means soft clustering (Futschik and Carlisle, 2005; Futschik, 2022). Low-expression genes were filtered out, and log_2_(FPKM + 1) values were z-score standardized. The optimal number of clusters (k = 8) was determined via the elbow method. Genes were assigned to clusters based on maximum membership values, and GO/KEGG enrichment was performed for each cluster.

### 2.5 Weighted gene co-expression network analysis

WGCNA was conducted in R (Zhang and Horvath, 2005; Langfelder and Horvath, 2008). Genes with low variance (<0.1) were excluded. A soft threshold of 6 was selected to achieve an approximately scale-free topology (R² ≈ 0.9) while maintaining sufficient mean connectivity. Adjacency matrices were converted to topological overlap matrices, and modules were identified using dynamic tree cutting (minModuleSize = 30, mergeCutHeight = 0.25). Modules were assigned colors, and eigengenes (first principal components) were correlated with developmental stages. Hub genes were identified based on intramodular connectivity (kWithin) and gene significance (GS) (Qu et al., 2022).

### 2.6 Candidate gene screening and integrative analysis

Core regulators were identified by intersecting genes with dynamic expression patterns (Mfuzz) and hub genes from WGCNA modules correlated with developmental stages, yielding an initial set of 200 candidates. These were refined using: (1) module membership (MM) ≥ 0.8; (2) functional annotations relevant to branching, axillary buds, hormone signaling, or transcriptional regulation; (3) consistent expression trends across natural stages. S representative genes, including ARF, IAA16, and SAUR36, were selected for qRT-PCR validation. From this initial candidate set, a small number of representative genes were selected for experimental validation based on their network connectivity, functional relevance to branching, and consistent temporal expression patterns.

### 2.7 Quantitative real-time PCR validation

#### 2.7.1 Validation of candidate gene expression patterns

qRT-PCR was performed on RNA from axillary buds at T0, T1, and T2 (n = 5) (Tang et al., 2023; Wang et al., 2023; Xie et al., 2024). cDNA synthesis used HiScript II Q RT SuperMix (+gDNA wiper). Reactions were run on a QuantStudio system with ChamQ Universal SYBR qPCR Master Mix. Primers annealed at 54°C, and 18S rRNA was the reference. Relative expression was calculated via 2^-^ΔΔCt. One-way ANOVA with Tukey’s *post-hoc* test (p < 0.05) assessed significance; three technical replicates were performed.

#### 2.7.2 Expression profiling of candidate genes under hormone treatment

FPKM values for the six candidate genes were extracted from the IAA treatment time-course (0–48 h), log_2_(FPKM + 1)-transformed, and visualized in R using ggplot2. Temporal expression profiles revealed rapid, stage-specific responses, highlighting their roles as key mediators in auxin signaling during axillary bud development (Wójcikowska and Gaj, 2017; Shen et al., 2014; Markakis et al., 2013; Ren et al., 2015).

## 3 Results

### 3.1 Transcriptome overview and quality assessment

High-throughput RNA sequencing of 15 axillary bud samples from *H. rhamnoides* across three developmental stages (T0, T1, and T2) generated 96.43 Gb of high-quality clean data, with an average of 6.43 Gb per sample. All libraries exhibited high sequencing quality, with Q30 values exceeding 94.05%. In addition, the low proportion of rRNA-derived reads indicated high RNA purity suitable for downstream transcriptomic analyses (**Table 1**). Clean reads were successfully aligned to the reference genome, with alignment rates ranging from 91.08% to 92.56% (mean 91.84%). CDS coverage ranged from 84.37% to 85.20%, indicating comprehensive gene representation adequate for robust quantitative analysis.

**Table 1:** Summary of RNA-seq data quality and mapping statistics for axillary bud samples across three developmental stages.

Sample name	Clean reads	Clean bases(bp)	Clean N(%)	Clean GC(%)	Clean Q20(%)	Clean Q30(%)	rRNA ratio(%)	Clean(%)
T0-1	40687498	6103124700	0.02;0.01	42.16;42.22	99.24;98.19	96.05;92.13	0.75	99.74
T0-2	40334430	6050164500	0.02;0.00	42.09;42.19	99.28;98.30	96.13;92.43	0.87	99.71
T0-3	40897096	6134564400	0.02;0.00	42.00;42.08	99.29;98.29	96.12;92.36	0.62	99.72
T0-4	41689172	6253375800	0.02;0.00	42.00;42.08	99.25;98.32	95.87;92.36	0.66	99.69
T0-5	49183102	7377465300	0.02;0.00	42.13;42.19	99.28;98.34	96.11;92.56	0.64	99.7
T1-1	41475256	6221288400	0.02;0.00	42.02;42.11	99.32;97.86	96.15;90.54	0.27	99.75
T1-2	42341758	6351263700	0.02;0.00	41.69;41.77	99.32;98.12	96.22;91.65	0.24	99.64
T1-3	39797046	5969556900	0.02;0.00	42.28;42.34	99.30;98.13	96.11;91.60	0.74	99.68
T1-4	40443382	6066507300	0.02;0.00	41.66;41.78	99.28;97.91	95.81;90.78	0.18	99.77
T1-5	42571336	6385700400	0.01;0.01	41.75;41.84	99.53;99.16	97.27;96.00	0.62	99.72
T2-1	39807218	5971082700	0.02;0.01	42.12;42.21	99.29;98.18	96.13;91.89	0.32	99.69
T2-2	47323048	7098457200	0.02;0.00	41.60;41.77	99.27;97.53	95.90;89.56	0.25	99.71
T2-3	48103950	7215592500	0.02;0.00	42.05;42.14	99.30;98.12	96.01;91.51	0.43	99.77
T2-4	41835072	6275260800	0.02;0.00	41.68;41.78	99.36;98.31	96.33;92.22	0.22	99.77
T2-5	46405806	6960870900	0.02;0.01	41.69;41.80	99.25;98.11	95.97;91.77	0.23	99.73

**Figure 2:**
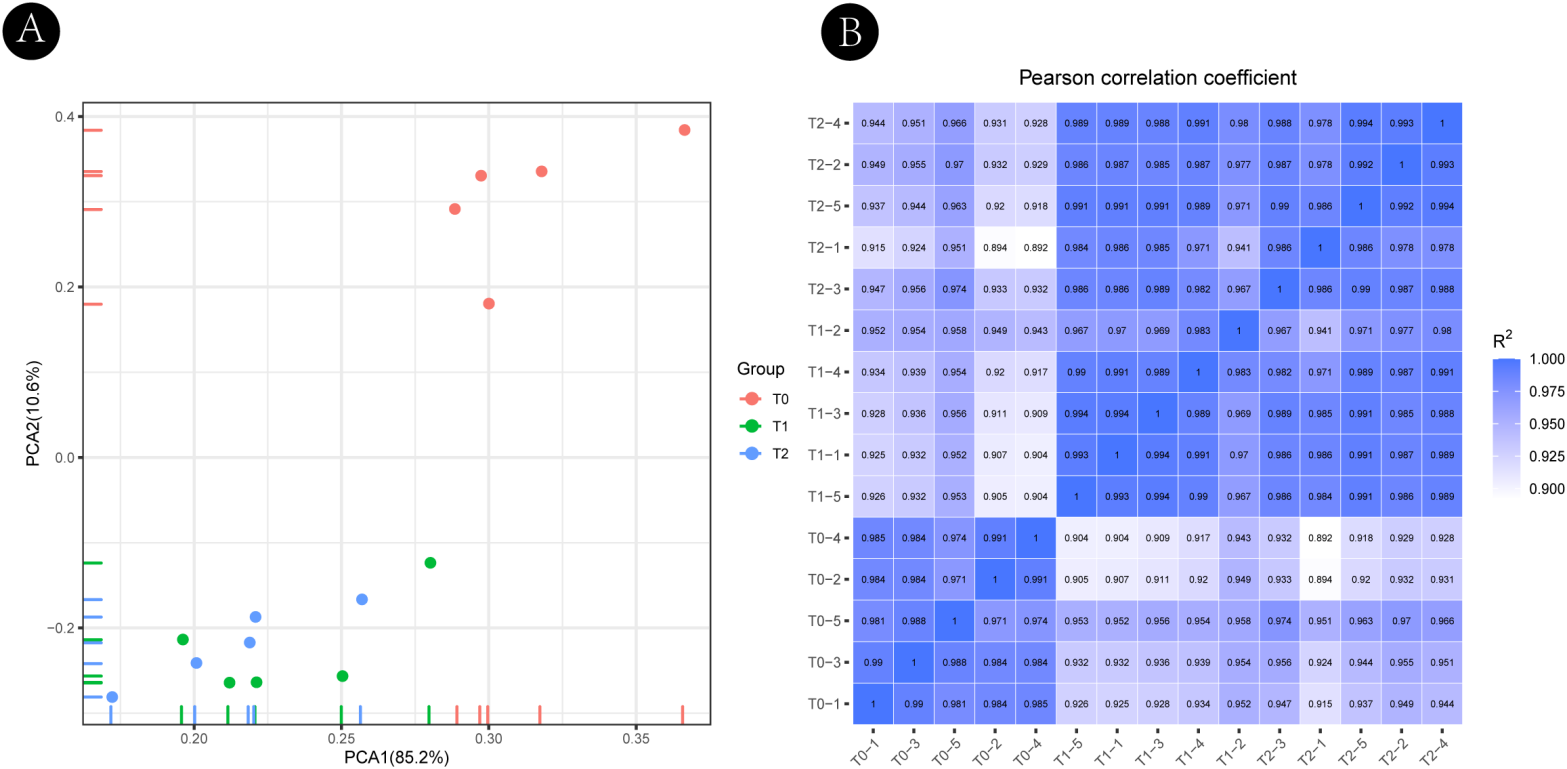
Transcriptomic profiling and sample reproducibility across axillary bud developmental stages in *Hippophae rhamnoides*. **(A)** Principal component analysis (PCA) of transcriptome profiles from axillary buds at three developmental stages: T0 (dormant), T1 (budburst), and T2 (branch elongation). Samples are grouped according to developmental stage. T0 forms a distinct cluster, whereas T1 and T2 exhibit partial overlap, reflecting major transcriptional reprogramming during bud activation followed by relative stabilization during early branch growth. **(B)** Heatmap of Pearson correlation coefficients (R^2^) among all biological replicates. The color gradient ranges from 0.900 to 1.000 (light to dark blue), indicating high correlation and strong reproducibility of the transcriptomic data within each developmental stage.

Principal component analysis (PCA) revealed clear separation of T0, T1, and T2 samples along the first two principal components, indicating substantial transcriptomic divergence among developmental stages (**Figure 2A**). Sample correlation analysis further demonstrated high reproducibility, with Pearson correlation coefficients exceeding 0.92 among biological replicates within each stage (**Figure 2B**).

For the 54 samples subjected to exogenous indole-3-acetic acid (IAA) treatments, sequencing quality metrics were equally robust, with Q30 scores >94%, an average mapping rate of 91.6%, and mean rRNA content of 0.5%. These results confirm the reliability of the sequencing data for subsequent analyses of transcriptional dynamics under hormonal treatment. Detailed quality statistics for IAA-treated samples are provided in **Supplementary Table S1**.

### 3.2 Endogenous auxin levels are stable during bud development

**Figure 3:**
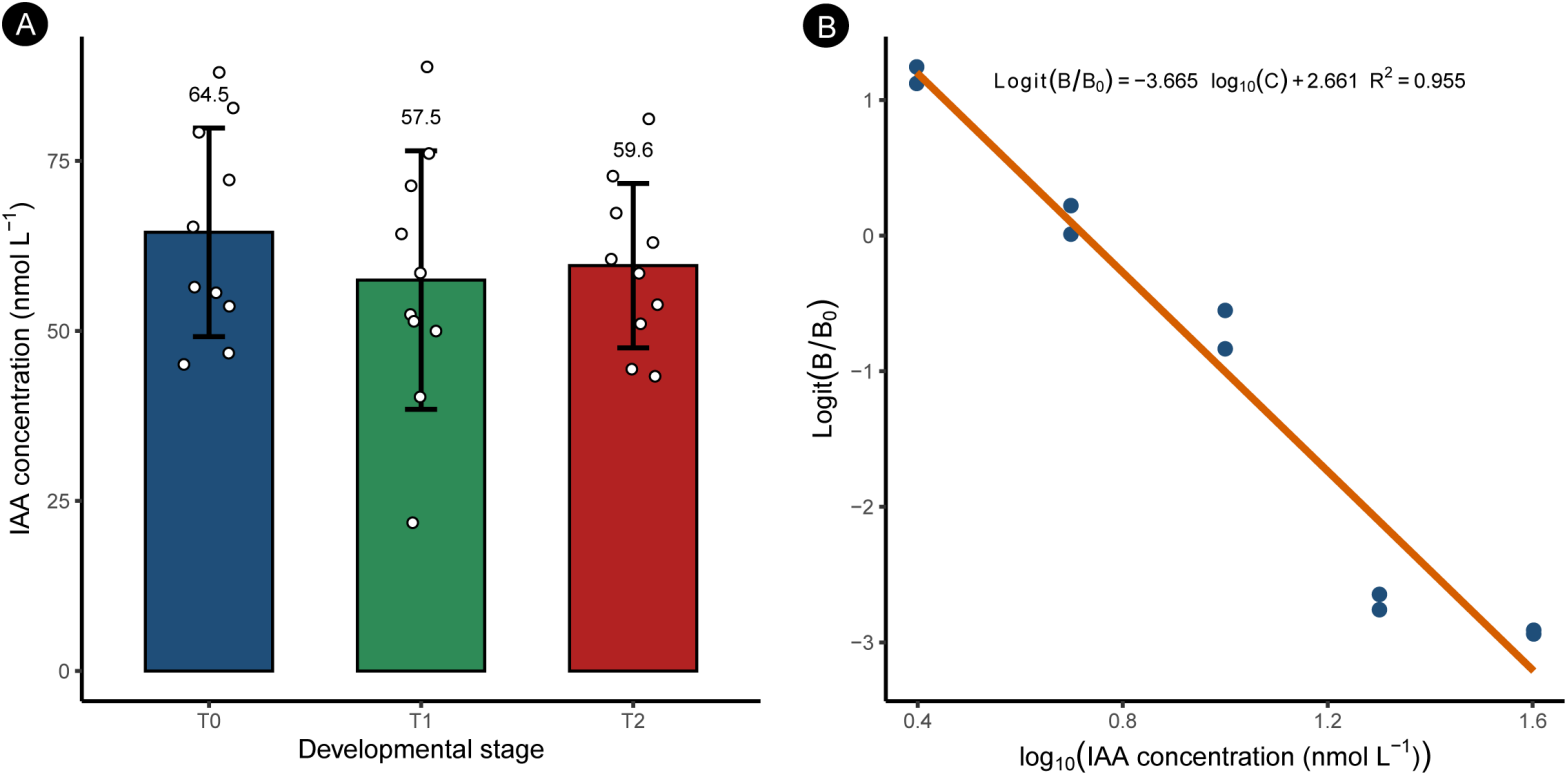
Quantification of endogenous indole-3-acetic acid (IAA) levels during axillary bud development. **(A)** Endogenous IAA concentrations in axillary buds at three developmental stages: dormancy (T0), budburst (T1), and elongation (T2), quantified using a competitive ELISA. Bars represent mean values, and error bars indicate standard deviation (SD) calculated from independent ELISA measurements derived from three biological replicates. Individual data points represent technical measurements. No statistically significant differences were detected among stages (one-way ANOVA, p > 0.05). **(B)** Standard curve for IAA quantification generated by competitive ELISA. Logit-transformed B/B_0_ values were plotted against the logarithm of IAA standard concentrations and fitted by linear regression. The regression equation and coefficient of determination (R²) are shown, indicating high assay reliability.

To examine whether axillary bud activation is accompanied by changes in endogenous auxin levels, we quantified indole-3-acetic acid (IAA) concentrations in axillary buds at three key developmental stages (T0–T2) using a competitive ELISA (**Figure 3**).

As shown in **Figure 3A**, the concentration of extractable IAA did not differ significantly among developmental stages (one-way ANOVA, p > 0.05). Mean IAA levels were 64.5 ± 15.3 nmol L^-^¹ in dormant buds (T0), 57.5 ± 19.0 nmol L^-^¹ during budburst (T1), and 59.6 ± 12.1 nmol L^-^¹ in elongating buds (T2). Despite the marked morphological transition from dormancy to active outgrowth, bulk tissue-level IAA concentrations remained relatively stable throughout axillary bud development.

The reliability of IAA quantification was supported by a well-fitted standard curve generated from the competitive ELISA assay (**Figure 3B**), showing a strong linear relationship between logit-transformed B/B_0_ values and the logarithm of IAA standard concentrations.

Together, these results indicate that axillary bud release in *H. rhamnoides* is not associated with a detectable change in bulk endogenous IAA levels under the conditions tested, suggesting that auxin-mediated regulation of bud activation may occur independently of changes in bulk hormone abundance. Raw ELISA measurements for endogenous IAA quantification, together with product information, are provided in **Supplementary Table S7**.

### 3.3 Transcriptomic dynamics during natural axillary bud development

#### 3.3.1 Differentially expressed genes across developmental stages

To systematically characterize transcriptional changes during axillary bud development in *H. rhamnoides*, we performed comparative transcriptome analyses across three key developmental stages (T0, T1, and T2). Sampling was conducted as illustrated in **Figure 1**, with five biological replicates per stage, each comprising pooled axillary buds from three branches of the same plant. DEGs were identified using thresholds of |log_2_FC| ≥ 1 and FDR < 0.05.

A total of 5,452 DEGs were identified between T0 and T1, accounting for approximately 49% of all expressed genes, indicating extensive transcriptional reprogramming during initial bud activation. Notably, the comparison between T2 and T0 also revealed the same total number of DEGs (5,452); however, the composition of upregulated and downregulated genes differed, indicating continued but reorganized transcriptional activity during branch elongation. In contrast, only 213 DEGs (67 upregulated, 146 downregulated) were detected between T1 and T2, suggesting transcriptional stabilization during subsequent branch elongation.

**Figure 4:**
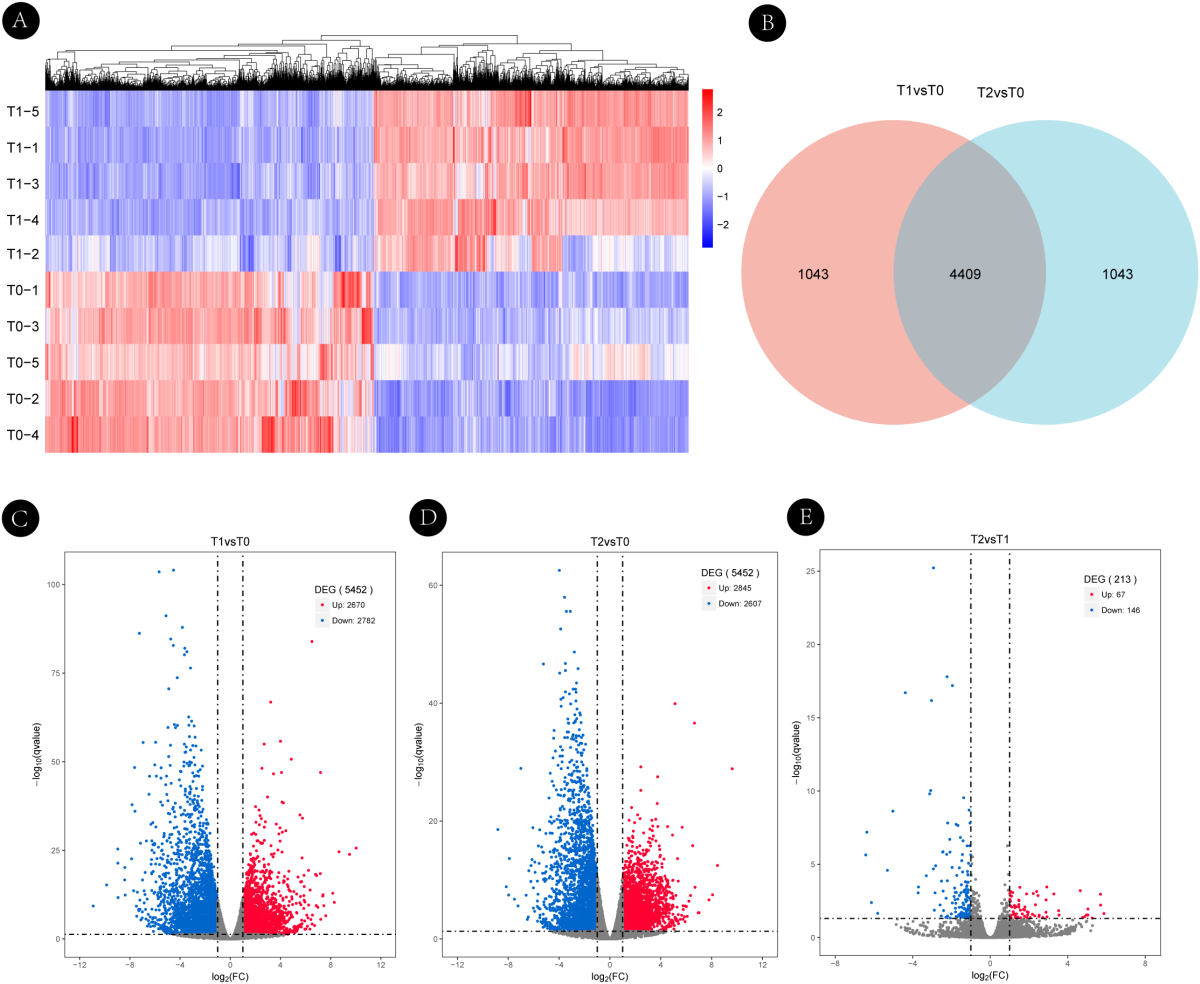
Transcriptomic dynamics and identification of differentially expressed genes (DEGs) during axillary bud development in *Hippophae rhamnoides*. **(A)** Hierarchical clustering heatmap of DEGs in the T1 vs. T0 comparison. Each row represents a gene, and each column represents a biological replicate. The color scale from blue to red indicates low to high gene expression levels (Z-score). **(B)** Venn diagram illustrating the overlap of DEGs between the T1 vs. T0 and T2 vs. T0 comparisons. A total of 4,409 DEGs were shared between the two comparisons, with 1,043 stage-specific DEGs in each comparison. **(C-E)** Volcano plots of DEGs for the pairwise comparisons between the three developmental stages: **(C)** T1 vs. T0, **(D)** T2 vs. T0, **(E)** T1 vs. T2. Significantly upregulated (red) and downregulated (blue) genes are highlighted (|log_2_FC| ≥ 1 and FDR < 0.05). Non-significant genes are shown in gray. The total number of DEGs for each comparison is 5,452 **(C)**, 5,452 **(D)**, and 213 **(E)**.

Stage-specific transcriptional shifts and high reproducibility among replicates were visualized through hierarchical clustering heatmaps and volcano plots (**Figures 4A, C–E**). The heatmap illustrated global expression patterns for the T1 vs. T0 comparison (**Figure 4A**), while volcano plots highlighted statistically significant DEGs across comparisons (**Figures 4C–E**). Detailed GO and KEGG enrichment results for pairwise comparisons are provided in the **Supplementary Figures**, with **Supplementary Figure S1** showing T0 vs. T1, **Supplementary Figure S2** showing T2 vs. T0, and **Supplementary Figure S3** showing T1 vs. T2. Venn diagram analysis further revealed 4,409 DEGs shared between T1 vs. T0 and T2 vs. T0, with 1,043 unique to each comparison, underscoring the coexistence of common and stage-specific transcriptional programs (**Figure 4B**). A complete list of differentially expressed genes from all pairwise comparisons is provided in **Supplementary Table S2**. These DEGs established a foundation for subsequent time-series clustering using Mfuzz and hub gene identification via WGCNA, enabling the capture of dynamic expression patterns and the identification of core regulators governing stage-specific developmental transitions.

#### 3.3.2 Functional enrichment analysis

To interpret the biological significance of these transcriptional changes, we performed GO and KEGG enrichment analyses for all identified DEGs.

In the T1 vs. T0 comparison, upregulated genes were significantly enriched in biological processes such as xylem development, organ morphogenesis, auxin response, and microtubule motor activity. The enrichment of xylem development-related pathways at the T0 and T1 stages is consistent with early axillary bud activation, during which vascular reconnection between the bud and the main stem is progressively established. This enrichment may reflect vascular-related developmental reprogramming during early bud activation, rather than necessarily indicating stem tissue contamination. KEGG analysis further indicated their involvement in plant hormone signal transduction and zeatin biosynthesis, suggesting a pronounced activation of hormonal signaling and cellular restructuring during the transition from dormancy to bud burst. In contrast, downregulated genes were associated with defense response, secondary metabolism, and oxidation–reduction processes, showing enrichment in phenylpropanoid biosynthesis, terpenoid biosynthesis, and the MAPK signaling pathway. These patterns reflect a metabolic shift from stress defense toward growth-oriented processes, highlighting the roles of hormonal reactivation and energy metabolism in bud release from dormancy.

For the T2 vs. T0 comparison, upregulated genes were primarily related to microtubule–kinetochore organization, chromatin assembly, and auxin transport. Corresponding KEGG pathways included plant hormone signal transduction, phenylpropanoid biosynthesis, and flavonoid biosynthesis, indicating active cell proliferation and differentiation during branch formation. Downregulated genes were mainly involved in plasma membrane composition, cell wall modification, and hormone responses, with significant enrichment in plant–pathogen interaction, MAPK signaling, and zeatin biosynthesis. It is noteworthy that the total number of DEGs in the T2 vs. T0 comparison (5,452) was identical to that in the T1 vs. T0 comparison, underscoring extensive transcriptional reprogramming across early and late developmental stages.

In the T1 vs. T2 comparison, where only a limited number of DEGs were identified (213), upregulated genes were enriched in abscisic acid (ABA) metabolism, osmotic stress response, and transcription factor activity. Downregulated genes were associated with auxin signaling, phospholipase C activity, and plasma membrane function. Key KEGG terms included plant hormone signal transduction, ABC transporters, and TCA cycle, implying a shift toward energy supply and environmental adaptation as buds progress from activation to elongation, with overall gene expression stabilizing during this phase.

Collectively, these enrichment results reveal stage-specific regulation of hormone signaling, metabolic activity, and cell proliferation during axillary bud development in *H. rhamnoides*. The initial bud activation stage (T0 → T1) is characterized by enhanced auxin- and cytokinin-related signaling and changes in energy- and growth-associated pathways, which are consistent with transcriptional reprogramming during bud outgrowth.

Detailed enrichment results for each comparison are provided in **Supplementary Figures S1**. These visualizations support stage-specific transcriptional dynamics and functional interpretations.

### 3.4 Temporal expression dynamics of key regulatory genes

#### 3.4.1 Stage-resolved temporal clustering analysis (Mfuzz)

**Figure 5:**
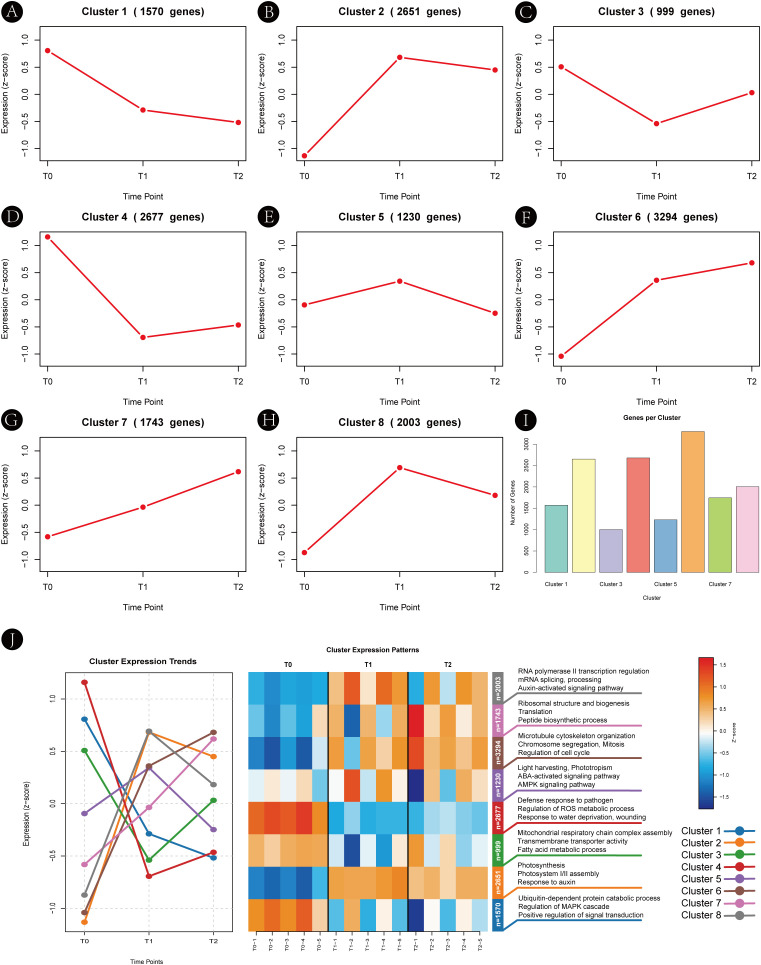
Temporal expression patterns and functional clustering of differentially expressed genes during axillary bud development in *Hippophae rhamnoides*. **(A–H)** Line graphs display the standardized expression trends (z-scores) for genes assigned to each of the eight Mfuzz clusters (Clusters 1–8) across three developmental time points (T0, T1, T2). The number of genes (n) in each cluster is indicated. **(I)** Bar plot showing the number of genes contained within each cluster. **(J)** Overview heatmap illustrating the overall expression patterns of all clusters, summarizing the dynamic transcriptional changes during the time course.

To investigate the temporal expression dynamics during axillary bud development in *H. rhamnoides*, we performed stage-resolved temporal clustering using the Mfuzz algorithm. This approach identified genes sharing similar expression trajectories, capturing coordinated regulatory programs underlying developmental transitions. Based on standardized expression profiles, genes were grouped into eight distinct clusters (Clusters 1–8) as shown in **Figure 5** and detailed in **Supplementary Table S3**.

The gene counts, expression patterns, and cluster classifications are summarized in **Supplementary Table S3** and visually represented in **Figures 5I, J**. Three major temporal expression modes were prominently represented: Cluster 8 (2,003 genes, ~18% of DEGs) exhibited an early-activation pattern, with expression rapidly increasing from T0 to T1, suggesting a pivotal role in bud initiation. Cluster 6 (3,294 genes, ~30%) displayed sustained activation, with gradual upregulation from T0 to T2, consistent with branch elongation and active cell proliferation. By contrast, Cluster 4 (2,677 genes, ~24%) showed a suppression–release pattern, characterized by transient downregulation from T0 to T1 followed by partial recovery, indicative of stress adaptation and energy metabolism in later developmental stages.

In summary, Mfuzz clustering effectively delineated temporal expression trajectories and revealed that distinct gene groups dominate at different developmental phases. These clusters capture dynamic transcriptional changes across developmental stages, providing a foundation for subsequent identification of hub genes and regulatory modules.

#### 3.4.2 Functional characterization of expression modules

To interpret the biological significance of these transcriptional changes, GO and KEGG enrichment analyses for all identified temporal clusters are shown in **Supplementary Figure S4**. The complete gene lists for each temporal expression cluster are provided in **Supplementary Table S3**.

The early-activation module (Cluster 8) was significantly enriched in genes involved in auxin signaling, transcriptional regulation, and cell fate determination. Expression peaked at T1 after a sharp increase from T0, followed by a slight decline at T2 (T0 < T1 > T2). Most candidate branching-related genes were concentrated in this cluster, underscoring its central role in initiating axillary bud outgrowth. Notably, the BRC1 homolog in *H. rhamnoides* was included in Cluster 8, exhibiting a rapid increase in expression from T0 to T1 during budburst, followed by a slight decrease at T2. This temporal pattern mirrors other early-activation genes, including key auxin-responsive regulators such as ARF, IAA16, and SAUR36, suggesting that BRC1 may act as an integrator of hormonal and transcriptional signals during early bud activation (Aguilar-Martínez et al., 2007; González-Grandío et al., 2013).

The sustained-activation module (Cluster 6) comprised genes related to cell division, microtubule organization, mitotic spindle formation, and ribosome biogenesis. Expression levels increased progressively from T0 to T2 (T0 < T1 < T2), supporting continuous branch growth and meeting the biosynthetic demands of developing buds.

In contrast, the suppression–release module (Cluster 4) was enriched in genes associated with reactive oxygen species (ROS) detoxification, ABA signaling, and mitochondrial energy metabolism. Expression decreased sharply from T0 to T1 and remained low through T2 (T0 > T1 ≈ T2), reflecting a transient suppression phase followed by metabolic adaptation during later stages of bud development.

Collectively, these results reveal a temporally coordinated transcriptional program in which hormonal signaling, transcriptional regulation, energy metabolism, and stress responses are orchestrated to regulate axillary bud outgrowth and branch formation. The inclusion of *BRC1* and other early auxin-responsive genes in Cluster 8 highlights a hub network potentially governing dormancy release and the initiation of branching. The correspondence between Mfuzz cluster patterns and functional annotations provides a robust framework for subsequent identification of hub genes and regulatory modules.

Although Cluster 8 represented the most prominent early-activation pattern, a small subset of genes in Cluster 2 also showed transient upregulation at T1, but did not contribute substantially to the final set of core branching-related candidates.

### 3.5 Weighted gene co-expression network reveals key modules associated with axillary bud development

#### 3.5.1 Network construction and module detection

To complement the time-series clustering analysis and further characterize coordinated gene expression patterns during axillary bud development, weighted gene co-expression network analysis (WGCNA) was performed using normalized FPKM values of all expressed genes. This approach clusters genes based on expression similarity across samples and facilitates the identification of co-expression modules with shared expression dynamics.

**Figure 6:**
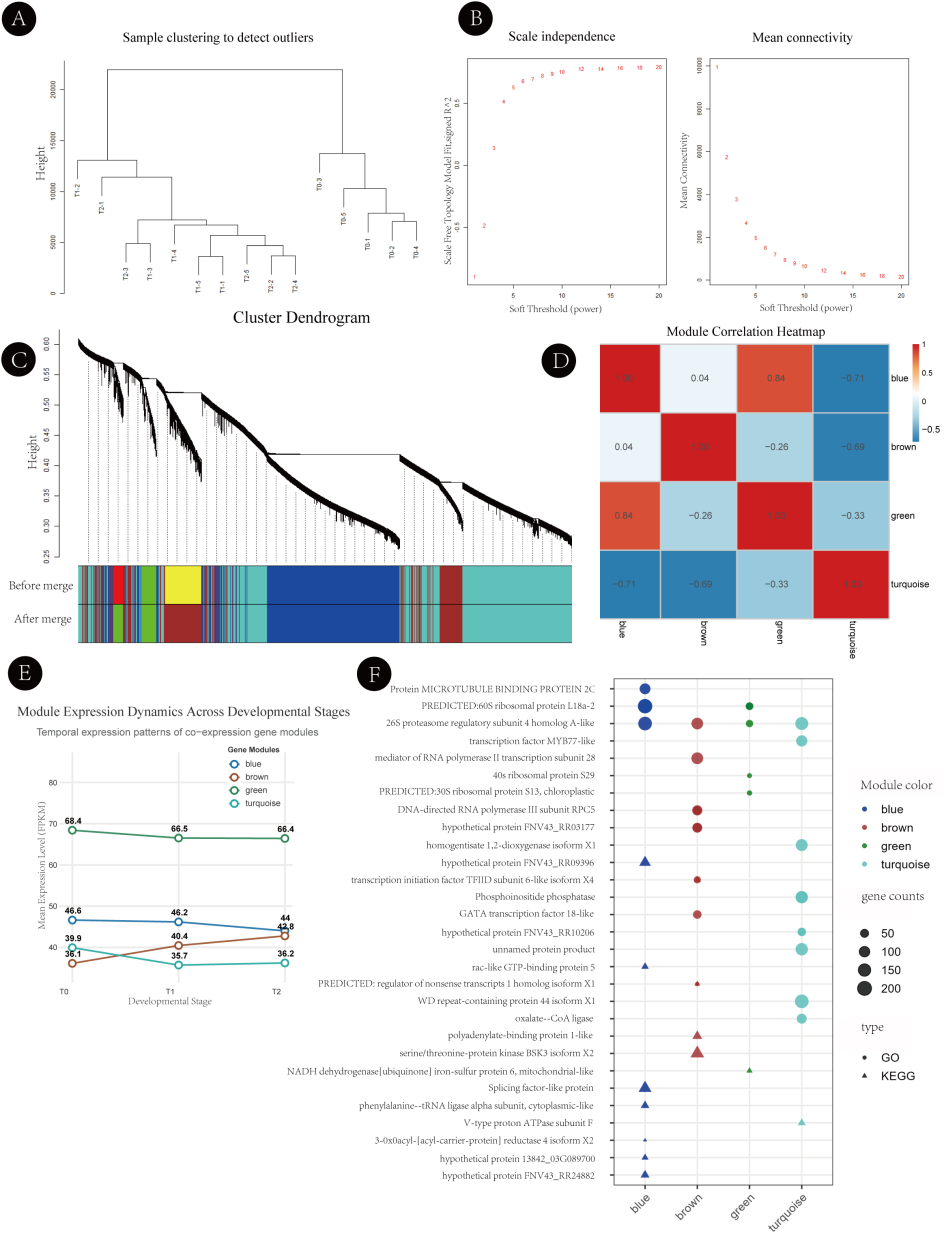
Weighted gene co-expression network analysis (WGCNA) of axillary bud development in *Hippophae rhamnoides*. **(A)** Sample clustering dendrogram used to detect potential outliers prior to network construction. **(B)** Analysis of network topology for soft-thresholding power selection: the left panel shows the scale-free topology fit index (scale independence), and the right panel shows mean connectivity under different soft-thresholding powers. **(C)** Hierarchical clustering dendrogram of genes based on the topological overlap matrix (TOM), with co-expression modules indicated by different colors. **(D)** Module eigengene correlation heatmap before (upper panel) and after (lower panel) merging of highly similar modules. **(E)** Eigengene expression profiles of the four merged co-expression modules (blue, brown, green, and turquoise) across three developmental stages (T0, T1, and T2). **(F)** Functional enrichment analysis and representative hub genes of the four key modules. Colored bars on the left indicate module identity. Dot size represents the number of genes enriched in each term, and dot color indicates the annotation database (GO or KEGG).

Genes with low expression variance (variance < 0.1) were excluded prior to network construction. Hierarchical clustering of samples revealed no apparent outliers, indicating that all samples were suitable for WGCNA (**Figure 6A**). A soft-thresholding power of 6 was selected to approximate a scale-free network topology (scale-free R² ≈ 0.9) while maintaining sufficient mean connectivity (**Figure 6B**). Based on the topological overlap matrix (TOM), hierarchical clustering combined with dynamic tree cutting identified multiple co-expression modules. After merging highly similar modules, four major modules—blue, brown, green, and turquoise—were retained for subsequent analyses (**Figure 6C**). Genes within each module exhibited highly correlated expression profiles, suggesting coordinated transcriptional behavior. The gene composition of each merged WGCNA module is provided in **Supplementary Table S4**.

#### 3.5.2 Stage-associated expression patterns of co-expression modules

To examine the relationship between co-expression modules and axillary bud developmental stages, module eigengene expression patterns were compared across T0, T1, and T2 (**Figure 6E**). The blue module showed relatively higher eigengene expression at T1 compared with T0 and T2, indicating an association with transcriptional changes occurring during bud activation. The brown module displayed a gradual increase in eigengene expression from T0 to T2, consistent with sustained transcriptional activity during bud outgrowth and branch development. In contrast, the turquoise module exhibited elevated eigengene expression at T2, suggesting potential involvement in later developmental processes. The green module maintained relatively stable expression across all stages, implying roles in basic cellular functions.

Correlation analysis among module eigengenes revealed distinct relationships among the identified modules (**Figure 6D**). Some 5modules showed positive correlations, whereas others exhibited negative correlations, reflecting differences in their overall expression trends across developmental stages. Collectively, these results indicate that WGCNA effectively captured structured co-expression modules with clear stage-associated expression patterns.

#### 3.5.3 Functional characterization and identification of hub genes

Functional enrichment analysis was conducted to explore the biological relevance of the four major modules (**Figure 6F**). The blue module was enriched in transcription factors (e.g., MYB77-like and GATA18-like) and ribosomal protein-related genes, suggesting a role in transcriptional regulation and translational capacity during axillary bud activation. The brown module was primarily enriched in translation-related processes and protein biosynthesis, consistent with its progressive upregulation during sustained bud growth. The green module was associated with redox regulation and amino acid metabolism, indicating potential involvement in maintaining cellular metabolic homeostasis. The turquoise module was enriched in protein phosphorylation and kinase activity, and included highly connected hub genes such as serine/threonine-protein kinase BSK3 and Rac-like GTP-binding protein 5, highlighting its potential role in signaling processes during later stages of bud development.

Overall, WGCNA identified biologically coherent co-expression modules and candidate hub genes associated with different stages of axillary bud development, providing a network-level perspective that complements the temporal expression patterns revealed by Mfuzz clustering.

### 3.6 Integrative analysis identifies core candidate regulators of axillary bud development

To identify central regulators underlying developmental transitions, we integrated the results of Mfuzz clustering and WGCNA network analysis. Hub genes were defined by high intramodular connectivity (kWithin > 0.9) and gene significance (GS > 0.6), indicating their central positions within stage-specific co-expression modules. Several transcription factor families—including *ARF, MYB*, and *NAC*—were identified as hub genes, potentially integrating hormonal, developmental, and stress-responsive pathways to coordinate axillary bud outgrowth.

By cross-referencing WGCNA hub genes with Mfuzz cluster assignments (T0→T1→T2), we pinpointed genes exhibiting both stage-specific expression patterns and central network positions as high-confidence regulatory hubs. Integration of early-activation clusters (Clusters 2 and 8) with the blue and turquoise WGCNA modules yielded a robust set of candidate genes showing marked upregulation during dormancy release (T0→T1) and sustained or increased expression during branch elongation (T2). Functional annotation indicated involvement in hormone signaling, transcriptional regulation, and cytoskeletal organization.

Notably, auxin-responsive genes such as *ARF, IAA16*, and *SAUR36*, which were highly connected within both the blue module and early-activation clusters, may contribute to auxin-associated transcriptional regulation during bud release. Other hub genes, including *NAC41, PP2C*, and *MAPKK2*, were associated with stress adaptation and signal transduction, reflecting a coordinated balance between developmental activation and environmental responsiveness.

Based on their consistent temporal expression, strong network connectivity, and functional relevance, these genes were designated as core candidate regulators of axillary bud development in *H. rhamnoides.* Their dual features—pronounced temporal dynamics and high intramodular connectivity—suggest that they represent key candidate nodes integrating hormonal signaling, transcriptional control, and cellular growth. These candidates were subsequently prioritized for experimental validation and hormone-response assays (Sections 3.7). A complete list of core branching-related hub genes identified through integrative analysis is provided in **Supplementary Table S5**.

### 3.7 Validation of candidate gene expression by qRT-PCR

#### 3.7.1 Expression patterns during natural developmental stages

**Figure 7:**
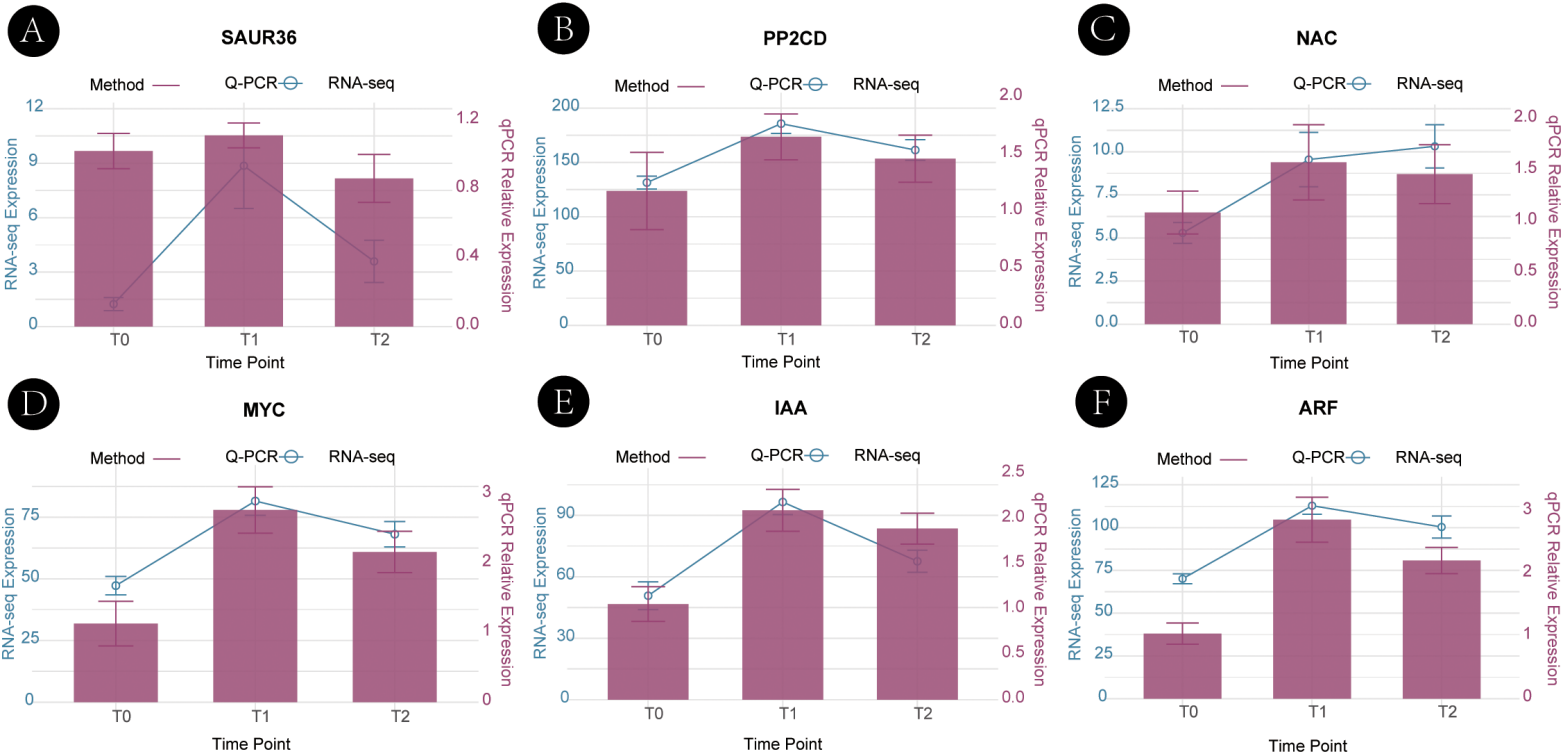
Validation of candidate gene expression patterns during natural developmental stages by qRT-PCR. Expression patterns of six candidate hub genes across three developmental stages (T0, T1, T2) are shown. Panels **A–F** correspond to SAUR36 **(A)**, PP2C **(B)**, NAC41 **(C)**, MYC **(D)**, IAA16 **(E)**, and ARF **(F)**. qRT-PCR results are shown as line graphs, and RNA-seq data are shown as bar plots for comparison. Error bars represent the standard deviations of three biological replicates.

To validate the reliability of the RNA-seq data, we examined the expression patterns of six key candidate genes—*ARF, IAA16, SAUR36, PP2C, MYC, and NAC41*—across three developmental stages (T0, T1, T2) using qRT-PCR. The qRT-PCR results were highly consistent with the RNA-seq data (**Figure 7**), confirming the accuracy of the transcriptomic analysis. Both methods revealed significant upregulation of these genes during the transition from bud dormancy (T0) to activation (T1), followed by stabilization or a slight decline during branch elongation (T2). Although minor differences in absolute expression levels were observed, these variations fell within acceptable ranges and did not affect the biological interpretation.

This strong concordance verifies the robustness of the RNA-seq dataset and provides experimental support for the potential regulatory roles of these genes in axillary bud development. Primer sequences, amplification conditions, and detailed qRT-PCR procedures are provided in the **Supplementary Materials**, Primer sequences and raw qRT-PCR data are provided in **Supplementary Table S6**.

#### 3.7.2 Expression responses to hormonal treatments

**Figure 8:**
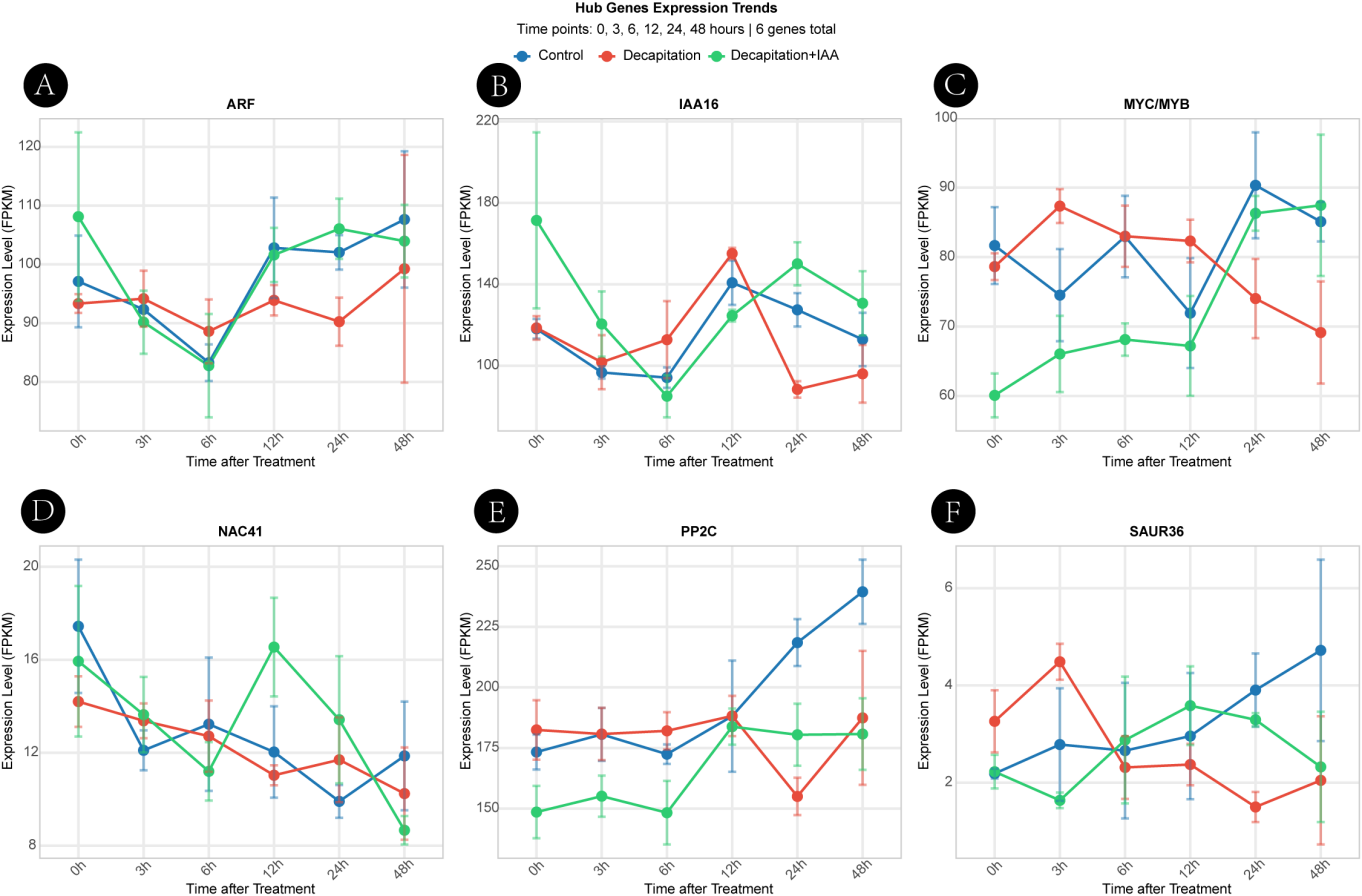
Expression responses of hub genes to decapitation and auxin treatment.**(A)** ARF, **(B)** IAA16, **(C)** MYC/MYB, **(D)** NAC41, **(E)** PP2C, and **(F)** SAUR36. Expression dynamics of six hub genes under three treatments—Control, Decapitation, and Decapitation + IAA—across a time course of 0, 3, 6, 12, 24, and 48 h. Gene expression levels (FPKM) are presented as line graphs, and error bars represent the standard deviation of three biological replicates.

To further investigate the roles of these genes in auxin-mediated bud outgrowth, we analyzed their expression dynamics following apical decapitation and exogenous IAA application (**Figure 8**). Decapitation significantly induced the expression of all six hub genes (*ARF, IAA16, SAUR36, PP2C, MYC, and NAC41*) within 3–48 hours, effectively releasing their transcriptional repression. This response pattern closely mirrored their activation during the natural T0-to-T1 transition, indicating that decapitation transcriptionally mimics the natural early activation process of axillary buds.

Importantly, exogenous IAA treatment partially or completely reversed the decapitation-induced upregulation, restoring gene expression to near-control levels. This “induction–rescue” response is consistent with auxin-mediated regulation of apical dominance and supports an association between apical auxin status and the transcriptional behavior of these candidate genes. Furthermore, distinct temporal response profiles were observed among genes: *ARF, IAA16, and SAUR36* showed rapid and transient induction, characteristic of early auxin-responsive elements, whereas *PP2C* and *NAC41* displayed delayed and sustained upregulation, suggesting roles in downstream signaling or hormonal crosstalk.

In summary, expression analyses under both natural development and hormone treatment conditions not only validate the transcriptomic findings but also identify a core set of auxin-responsive regulators crucial for axillary bud release in *H. rhamnoides*. These results establish a mechanistic framework for understanding the molecular regulation of branching architecture in woody plants.

## 4 Discussion

**Figure 9:**
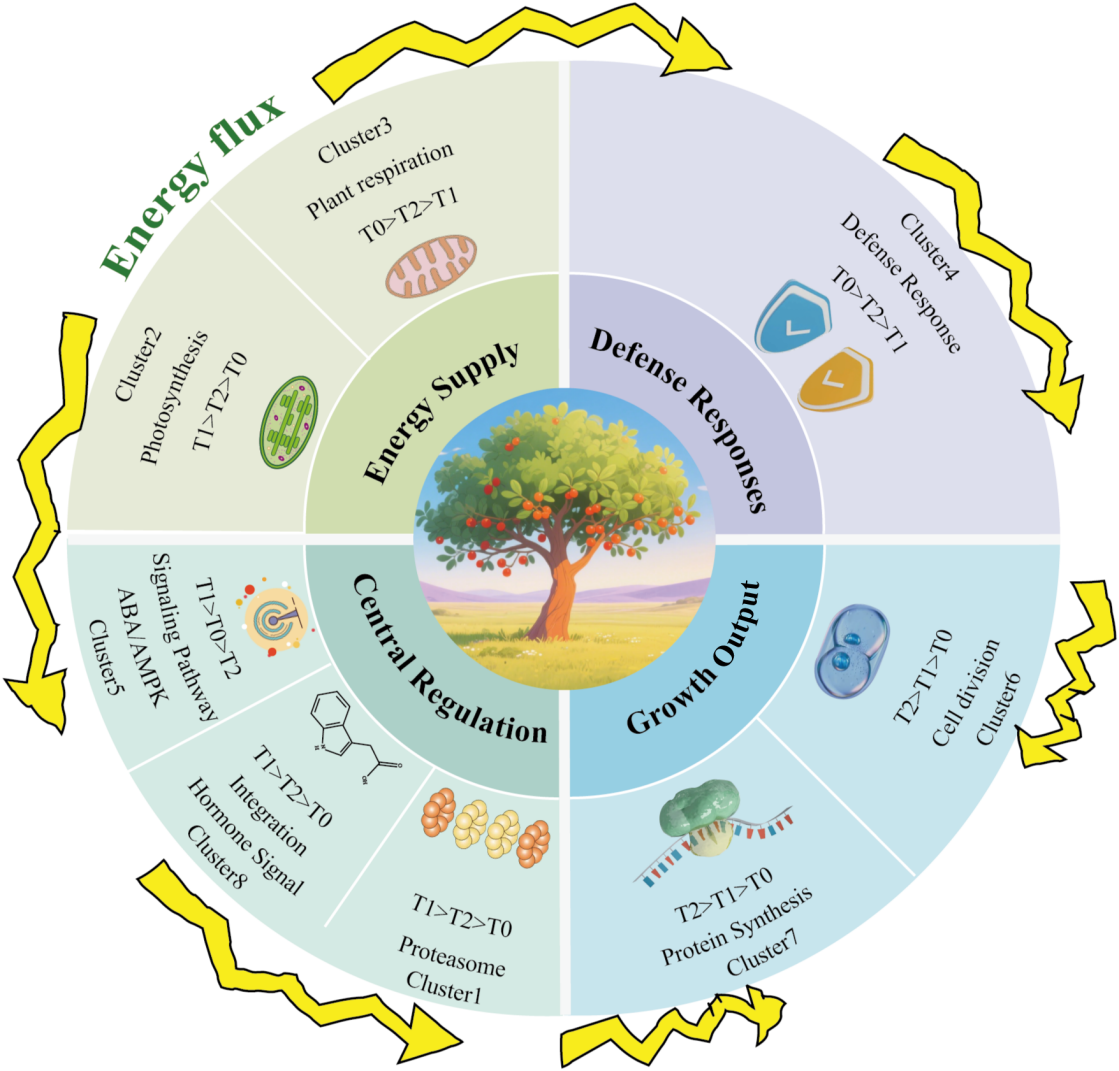
Integrated model of transcriptional and functional modularity during axillary bud development in *Hippophae rhamnoides*. The model summarizes four core functional categories: Energy Supply (e.g., respiration in Cluster 3), Defense Responses (Cluster 4), Central Regulation (e.g., proteasome activity in Cluster 1 and signaling in Cluster 5), and Growth Output (e.g., protein synthesis in Cluster 7 and cell division in Cluster 6). Arrows indicate proposed regulatory relationships or temporal transitions between modules across developmental stages (T0, T1, T2), illustrating the shift from dormancy to active growth.

The activation of axillary buds and their subsequent development into branches represent key determinants of shoot architecture in woody plants and are governed by complex interactions among hormonal, genetic, and metabolic factors (Domagalska and Leyser, 2011; Wang et al., 2018). In this study, we combined stage-resolved transcriptomic profiling, time-series clustering, and weighted gene co-expression network analysis (WGCNA) to systematically characterize the transcriptional landscape underlying the transition from bud dormancy to branch formation in *H. rhamnoides*. Together, these analyses support an integrative regulatory framework (**Figure 9**) in which axillary bud activation is associated with a coordinated shift from a dormant, resource-conserving state toward an active growth-executing state, consistent with recent models emphasizing the integration of hormonal and metabolic signals during shoot branching (Barbier et al., 2019).

Consistent with this framework, temporal expression analysis revealed distinct phases during axillary bud development. Genes in Cluster 8, which were significantly enriched in hormone signaling components and transcriptional regulators, exhibited rapid upregulation during the T0→T1 transition, indicating an early transcriptional activation phase associated with dormancy release. By contrast, Cluster 6 genes showed progressively increasing expression from T0 to T2 and were predominantly associated with cell proliferation and structural growth, consistent with their roles in sustaining branch elongation. In parallel, Cluster 4 and related modules displayed transient suppression followed by stabilization, suggesting dynamic adjustment of energy metabolism, redox homeostasis, and abscisic acid–related stress responses during developmental progression. Together, these temporal patterns highlight a sequential organization of transcriptional programs accompanying axillary bud activation and outgrowth.

**Figure 10:**
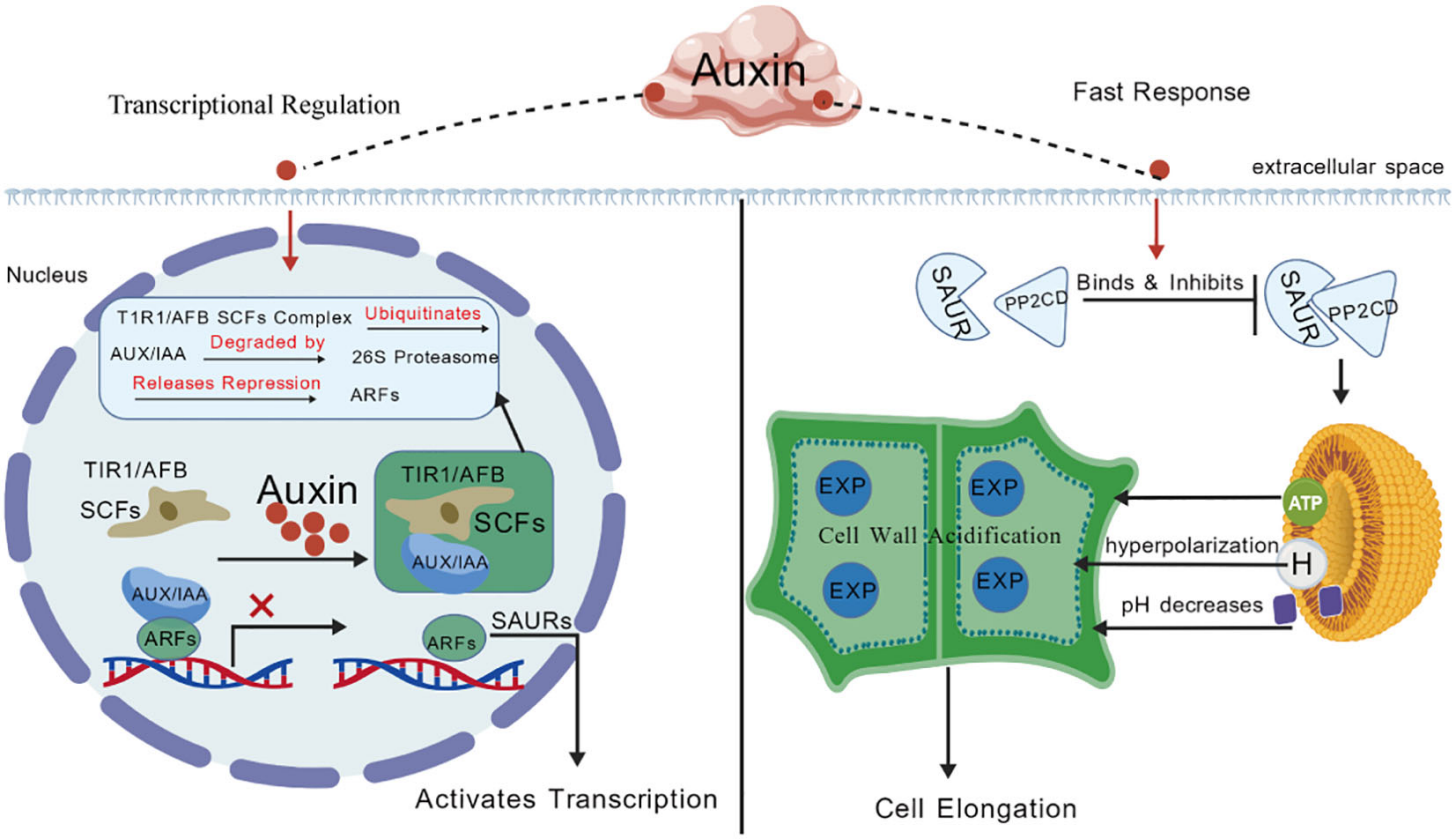
Shematic representation of the auxin signaling pathway involved in axillary bud activation. In the nucleus, auxin binds to the TIR1/AFB receptors in the SCF complex, promoting ubiquitination and degradation of AUX/IAA repressors via the 26S proteasome. This releases ARF transcription factors from repression, enabling activation of downstream gene expression. The model highlights the fast transcriptional response mediated by this pathway, which regulates key processes such as cell elongation and bud outgrowth.

Auxin-related transcriptional regulation emerged as a prominent feature during the early activation phase. Several candidate genes, including *ARF, IAA16*, and *SAUR36*, displayed rapid and transient induction, a characteristic signature of early auxin-responsive genes (Powers and Strader, 2020). This expression behavior is consistent with the canonical auxin signaling pathway (**Figure 10**), in which auxin promotes the degradation of *AUX/IAA* repressors, thereby releasing ARF transcription factors to activate downstream target genes involved in cell expansion and growth (Dharmasiri et al., 2005; Kepinski and Leyser, 2005; Gomes and Scortecci, 2021). in which auxin promotes the degradation of *AUX/IAA* repressors, thereby releasing ARF transcription factors to activate downstream target genes involved in cell expansion and growth (Dharmasiri et al., 2005; Kepinski and Leyser, 2005). In contrast, genes such as *PP2C* and *NAC41* exhibited delayed and more sustained expression changes, suggesting potential roles in downstream signal modulation or integration with additional hormonal and stress-related pathways.

Notably, decapitation and exogenous auxin application experiments further support a regulatory association between apical auxin status and the transcriptional behavior of these candidate genes. Removal of the apical bud was accompanied by increased expression of multiple hub genes, whereas subsequent application of exogenous IAA partially attenuated this response. This “release-and-rescue” expression pattern is consistent with established models of auxin-mediated apical dominance and competitive bud growth (Prusinkiewicz et al., 2009; Balla et al., 2016). However, because bulk IAA levels remained relatively stable across developmental stages, these findings suggest that transcriptional reprogramming during bud activation may reflect changes in auxin signaling output, transport dynamics, or cellular sensitivity rather than alterations in total hormone abundance.

Integration of Mfuzz-based temporal clustering with WGCNA further clarified the regulatory architecture underlying axillary bud development. The early activation cluster overlapped extensively with the blue WGCNA module, which was enriched in hormone-responsive genes and transcription factors, underscoring its potential role in coordinating the initial release from dormancy. In contrast, the turquoise module, which peaked during later stages, was associated with protein phosphorylation, kinase activity, and signaling processes linked to sustained growth and differentiation. These complementary analyses illustrate how temporally dynamic gene expression patterns are embedded within broader co-expression networks that organize stage-specific developmental functions.

Taken together, the results of this study support a modular view of axillary bud development in *H. rhamnoides*, in which distinct but interconnected transcriptional programs operate at successive stages of development. Early phases are characterized by hormone-responsive transcriptional activation and energy reallocation, whereas later phases involve sustained growth-related processes and metabolic adjustment. By integrating stage-resolved transcriptomics, network analysis, and targeted experimental validation, this work provides a systems-level framework that is consistent with current models of shoot branching regulation in woody plants and highlights candidate regulatory genes for future functional investigation.

## 5 Conclusion

This study systematically investigated the molecular regulation of axillary bud development in *H. rhamnoides* through an integrated approach combining stage-resolved transcriptomics and weighted gene co-expression network analysis. Our findings support a coordinated transcriptional framework in which distinct gene modules are sequentially associated with the transition from bud dormancy to branch formation. The early activation phase is characterized by auxin-responsive signaling patterns involving key regulators including *ARF, IAA16, and SAUR36*, while subsequent stages involve transcriptional reprogramming associated with *NAC, MYC*, and *PP2C/PP2CD* signaling phosphatases, along with stress adaptation mechanisms mediated by *PP2C* and other signaling components.

Notably, we identified the blue and turquoise modules from WGCNA as core regulatory units associated with bud activation and branch elongation, respectively. These modules contain hub genes that collectively exhibit a putative “release-and-rescue” transcriptional pattern in relation to apical auxin status, consistent with long-standing models of auxin-dependent apical dominance. Rather than indicating direct causal control, this modular organization suggests that coordinated transcriptional regulation underlies developmental transitions during axillary bud outgrowth.

Taken together, the integrative framework established in this study advances a systems-level perspective on shoot branching regulation in woody plants and highlights candidate regulatory genes for future functional investigation. By delineating stage-associated transcriptional modules and their network connectivity, this work provides a molecular resource for understanding plant architecture and offers potential targets for improving branching traits in perennial species.

## Data Availability

The datasets presented in this study can be found in onlinerepositories. The names of the repository/repositories and accessionnumber(s) can be found below: https://www.cncb.ac.cn/, GSA: CRA031583.
