# Supplementary material for: Time-resolved transcriptomic analysis reveals key regulatory genes and auxin-responsive networks underlying axillary bud branching in Hippophae rhamnoides
Source: Front Plant Sci. 2026 Mar 3;17:1746947. doi: 10.3389/fpls.2026.1746947 (PMC12993749; doi:10.3389/fpls.2026.1746947)
Supplement: Supplementary file 1 [file DataSheet1.pdf]

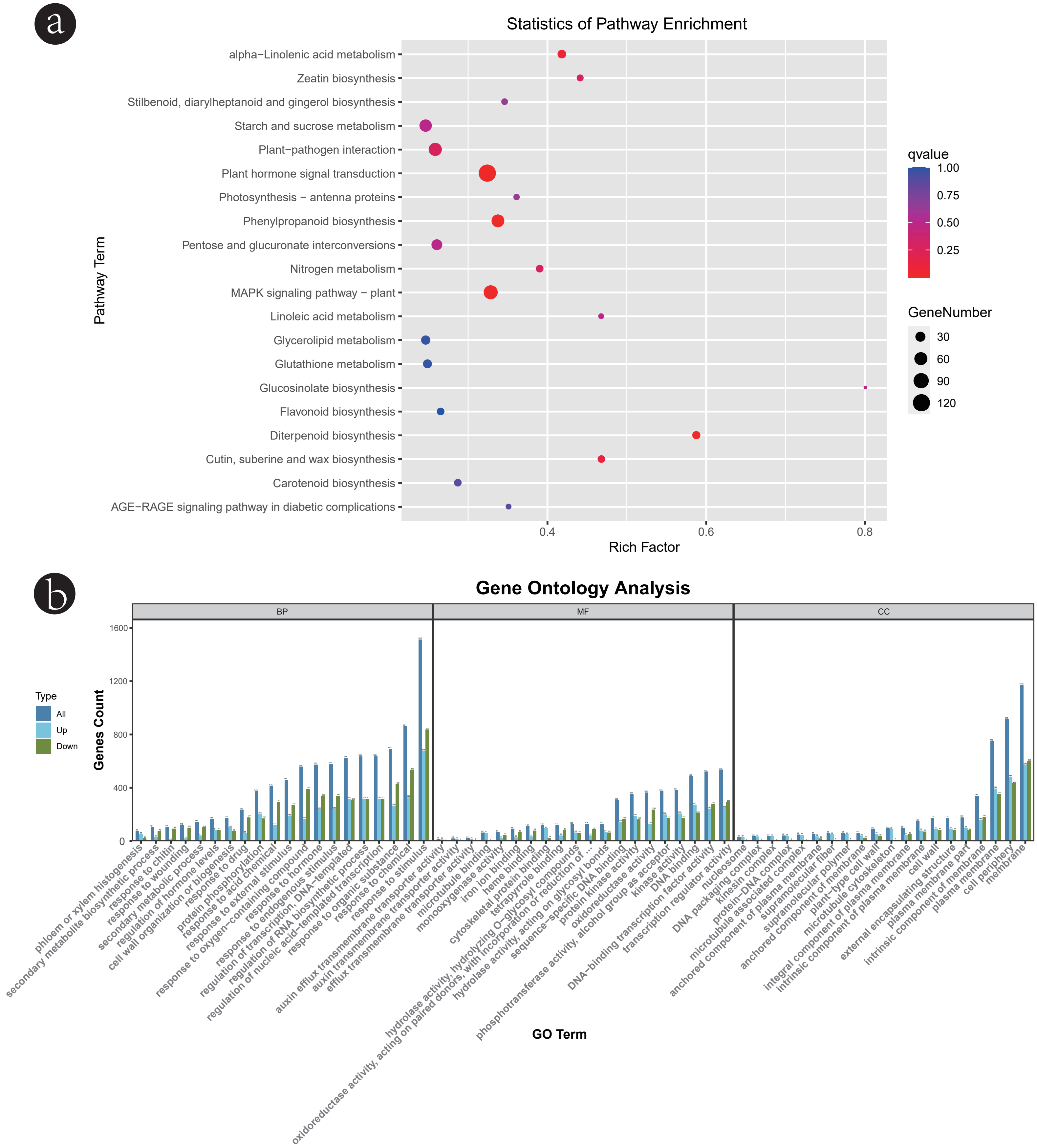

Figure S1. Gene Ontology (GO) and KEGG pathway enrichment analyses of differentially expressed genes between T1 and T0 stages.

(a) Gene Ontology (GO) enrichment analysis of differentially expressed genes (DEGs) identified in the T1 vs. T0 comparison, showing significantly enriched GO terms across the biological process, molecular function, and cellular component categories.
(b) KEGG pathway enrichment analysis of DEGs from the T1 vs. T0 comparison, highlighting significantly overrepresented metabolic and signaling pathways during early axillary bud activation.

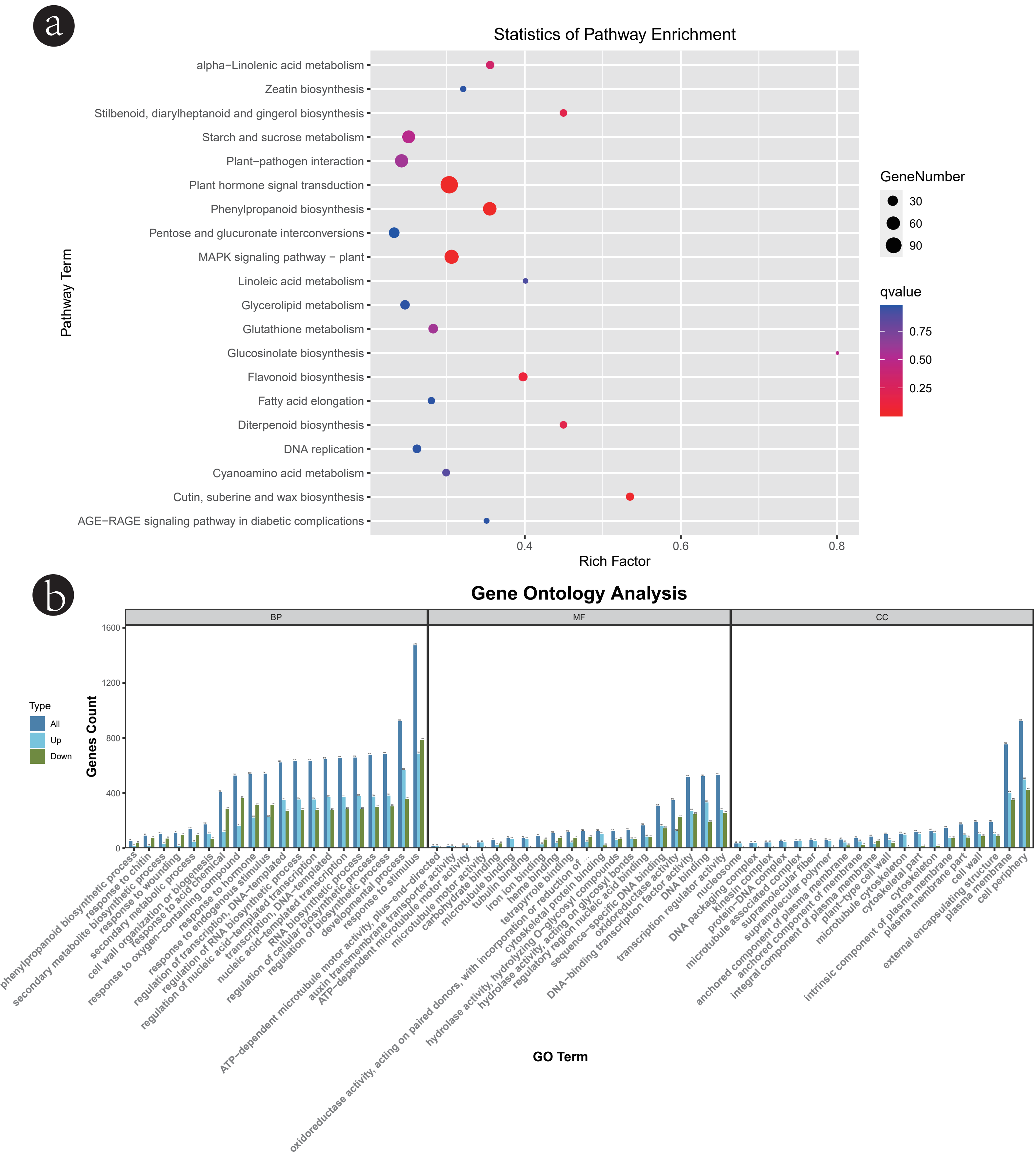

Figure S2. Gene Ontology (GO) and KEGG pathway enrichment analyses of differentially expressed genes between T2 and T0 stages.

(a) Gene Ontology (GO) enrichment analysis of differentially expressed genes (DEGs) identified in the T2 vs. T0 comparison, showing significantly enriched GO terms across the biological process, molecular function, and cellular component categories.
(b) KEGG pathway enrichment analysis of DEGs from the T2 vs. T0 comparison, highlighting overrepresented metabolic and signaling pathways associated with axillary bud outgrowth and early branch development.

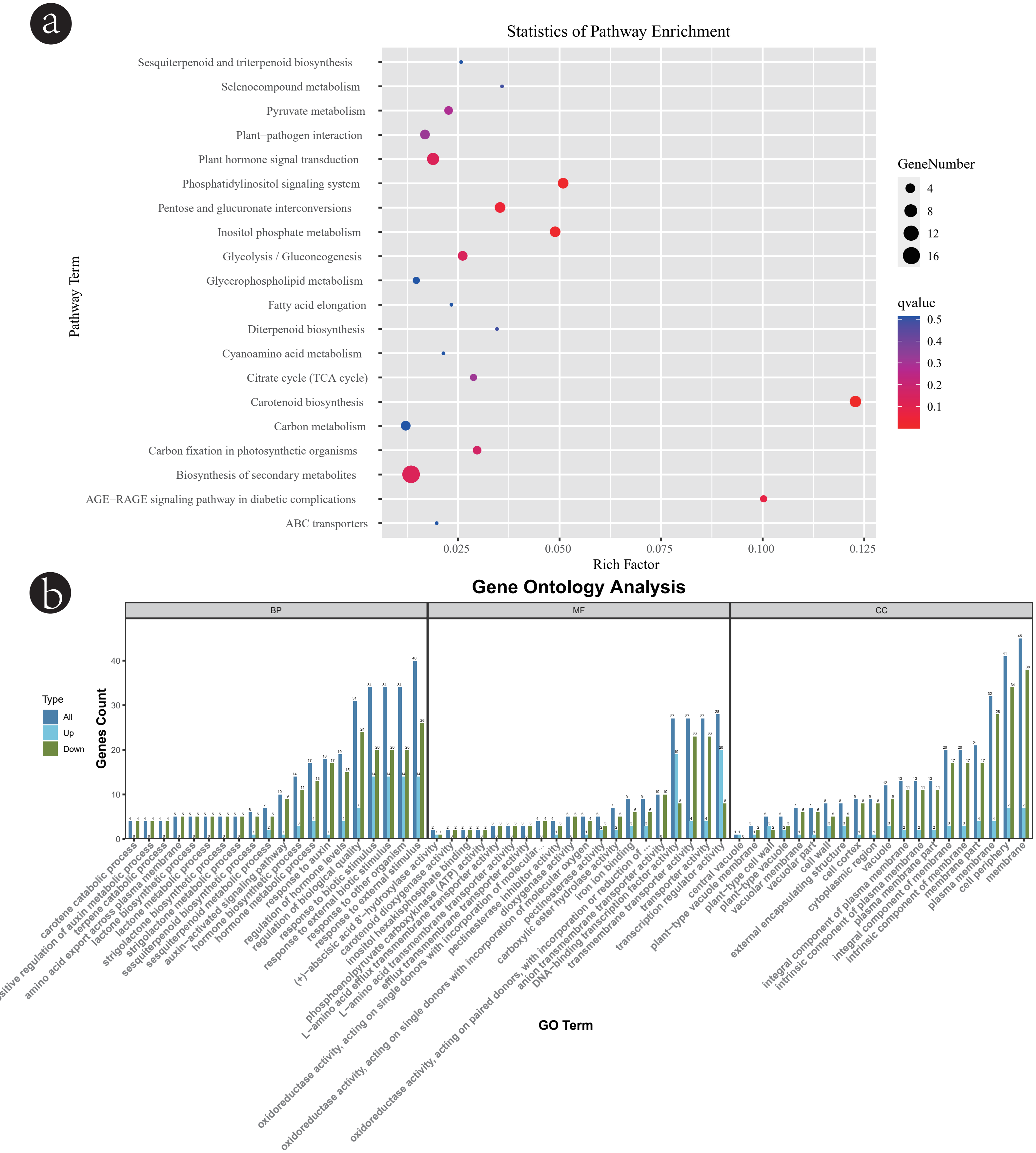

Figure S3. Gene Ontology (GO) and KEGG pathway enrichment analyses of differentially expressed genes between T2 and T1 stages.

(a) Gene Ontology (GO) enrichment analysis of differentially expressed genes (DEGs) identified in the T2 vs. T1 comparison, showing significantly enriched GO terms across the biological process, molecular function, and cellular component categories.

(b) KEGG pathway enrichment analysis of DEGs from the T2 vs. T1 comparison, highlighting pathways associated with the transition from bud activation to sustained outgrowth and branch elongation.

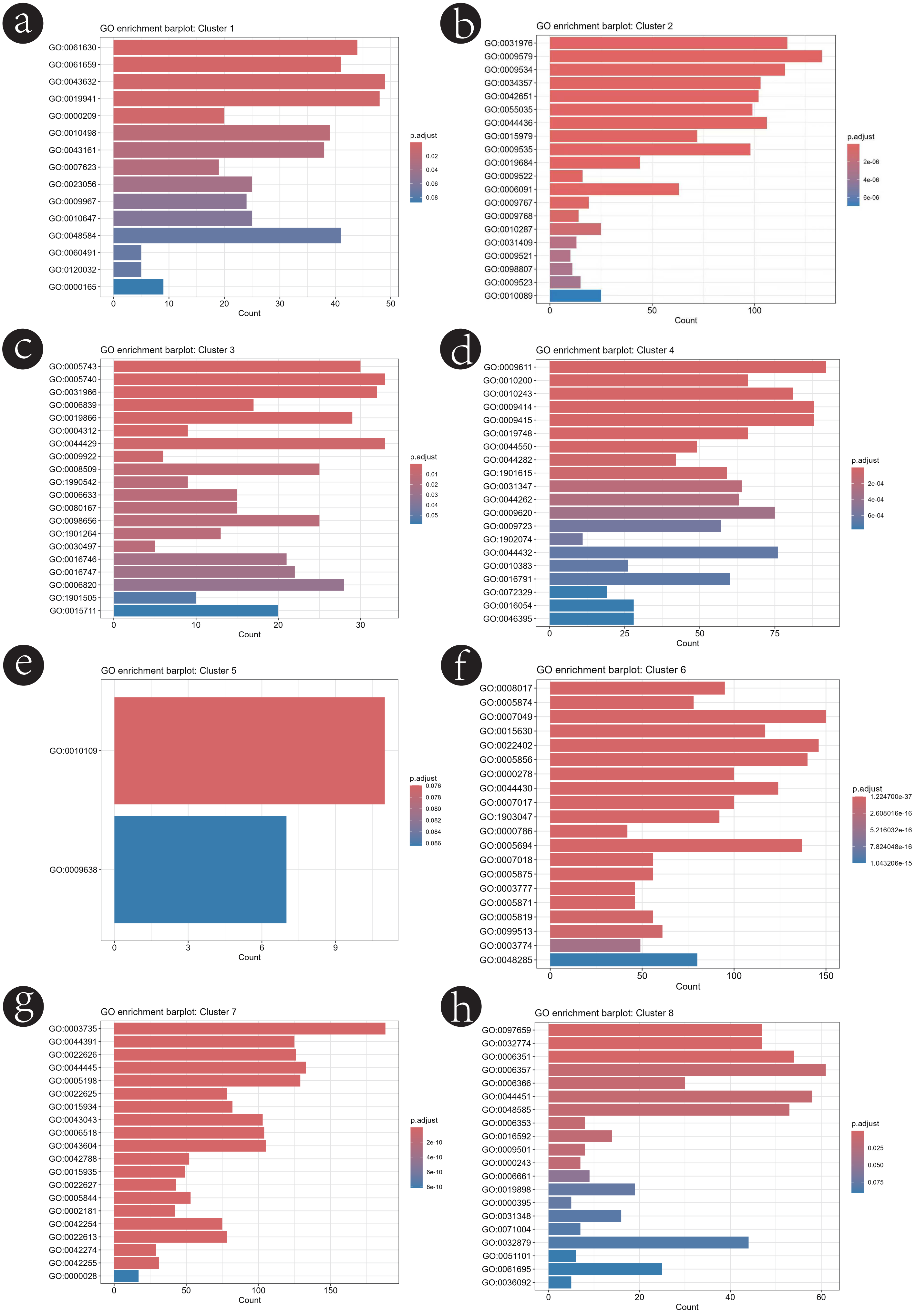
a
GO enrichment barplot: Cluster 1
GO:0061630
GO:0061659
GO:0043632
GO:0019941
GO:0000209
GO:0010498
GO:0043161
GO:0007623
GO:0023056
GO:0009967
GO:0010647
GO:0048584
GO:0060491
GO:0120032
GO:0000165
0 10 20 30 40 50
Count
p.adjust
0.02
0.04
0.06
0.08
b
GO enrichment barplot: Cluster 2
GO:0031976
GO:0009579
GO:0009534
GO:0034357
GO:0042651
GO:0055035
GO:0044436
GO:0015979
GO:0009535
GO:0019684
GO:0009522
GO:0006091
GO:0009767
GO:0009768
GO:0010287
GO:0031409
GO:0009521
GO:0098807
GO:0009523
GO:0010089
0 50 100
Count
p.adjust
2e-06
4e-06
6e-06
c
GO enrichment barplot: Cluster 3
GO:0005743
GO:0005740
GO:0031966
GO:0006839
GO:0019866
GO:0004312
GO:0044429
GO:0009922
GO:0008509
GO:1990542
GO:0006633
GO:0080167
GO:0098656
GO:1901264
GO:0030497
GO:0016746
GO:0016747
GO:0006820
GO:1901505
GO:0015711
0 10 20 30
Count
p.adjust
0.01
0.02
0.03
0.04
0.05
d
GO enrichment barplot: Cluster 4
GO:0009611
GO:0010200
GO:0010243
GO:0009414
GO:0009415
GO:0019748
GO:0044550
GO:0044282
GO:1901615
GO:0031347
GO:0044262
GO:0009620
GO:0009723
GO:1902074
GO:0044432
GO:0010383
GO:0016791
GO:0072329
GO:0016054
GO:0046395
0 25 50 75
Count
p.adjust
2e-04
4e-04
6e-04
e
GO enrichment barplot: Cluster 5
GO:0010109
GO:0009638
0 3 6 9
Count
p.adjust
0.076
0.078
0.080
0.082
0.084
0.086
f
GO enrichment barplot: Cluster 6
GO:0008017
GO:0005874
GO:0007049
GO:0015630
GO:0022402
GO:0005856
GO:0000278
GO:0044430
GO:0007017
GO:1903047
GO:0000786
GO:0005694
GO:0007018
GO:0005875
GO:0003777
GO:0005871
GO:0005819
GO:0099513
GO:0003774
GO:0048285
0 50 100 150
Count
p.adjust
1.224700e-37
2.608016e-16
5.216032e-16
7.824048e-16
1.043206e-15
g
GO enrichment barplot: Cluster 7
GO:0003735
GO:0044391
GO:0022626
GO:0044445
GO:0005198
GO:0022625
GO:0015934
GO:0043043
GO:0006518
GO:0043604
GO:0042788
GO:0015935
GO:0022627
GO:0005844
GO:0002181
GO:0042254
GO:0022613
GO:0042274
GO:0042255
GO:0000028
0 50 100 150
Count
p.adjust
2e-10
4e-10
6e-10
8e-10
h
GO enrichment barplot: Cluster 8
GO:0097659
GO:0032774
GO:0006351
GO:0006357
GO:0006366
GO:0044451
GO:0048585
GO:0006353
GO:0016592
GO:0009501
GO:0000243
GO:0006661
GO:0019898
GO:0000395
GO:0031348
GO:0071004
GO:0032879
GO:0051101
GO:0061695
GO:0036092
0 20 40 60
Count
p.adjust
0.025
0.050
0.075

Figure S4. Gene Ontology (GO) enrichment analysis of genes in Mfuzz-derived temporal expression clusters.

(a – h) GO enrichment bar plots of genes assigned to eight Mfuzz-based temporal expression clusters (Clusters 1 – 8). In each panel, enriched GO terms are shown as GO term identifiers (GO IDs) on the y-axis, and the x-axis represents the number of genes (Count) associated with each GO term. Bar colors indicate the adjusted p-values (p.adjust), reflecting the statistical significance of enrichment.

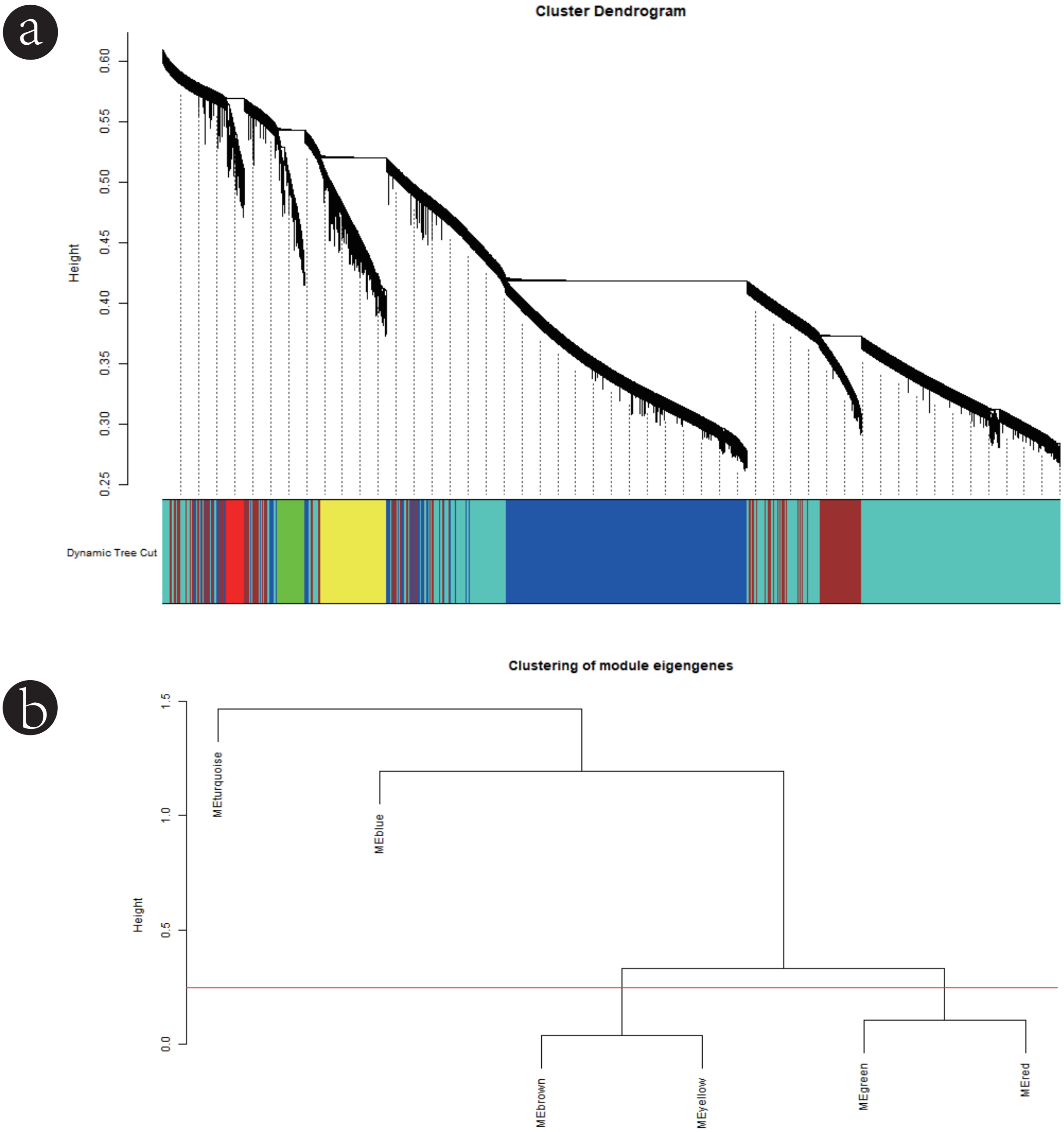

Figure S5. Weighted gene co-expression network analysis (WGCNA) of axillary bud transcriptomes.

(a) Hierarchical clustering dendrogram of genes based on topological overlap, showing initial co-expression modules identified by the dynamic tree cut algorithm before module merging. Colored bars below the dendrogram represent the original WGCNA modules prior to merging.
(b) Hierarchical clustering of WGCNA module eigengenes, illustrating the relationships among modules and providing the basis for subsequent module merging. The red horizontal line indicates the threshold used to define module similarity.
